# Floquet topological states in time-varying metasurfaces

**DOI:** 10.1126/sciadv.adx9025

**Published:** 2025-09-26

**Authors:** Qian Ma, Jian Wei You, Long Chen, Ze Gu, Shi Long Qin, Zhihao Lan, Tie Jun Cui

**Affiliations:** ^1^ State Key Laboratory of Millimeter Waves and Institute of Electromagnetic Space, Southeast University, Nanjing, China.; ^2^ Department of Electronic and Electrical Engineering, University College London, London, UK.

## Abstract

Floquet systems offer a nonequilibrium periodic platform to explore previously unknown phases of matter, attracting interest in condensed matter physics, quantum physics, and photonics. Most existing research focuses on linear, closed systems subjected to spatially periodic driving. Here, we present a programmable time-varying metasurface as a platform for investigating Floquet topological states. Our findings indicate that a topological transition occurs in response to modulation frequency increases, producing anomalous edge states with chirality within Floquet harmonic bandgaps. The open nature of the metasurface facilitates harmonic generation via Floquet edge states. Experimentally, we confirm the existence of Floquet harmonic bands and the robust unidirectional propagation of these edge states. In addition, we propose a large-scale coding scheme to dynamically reconfigure propagation paths through programmable binary states of the meta-units. The system not only provides a unique platform to explore interesting physics, it can also offer opportunities for diverse applications.

## Teaser

Floquet topological states and harmonic generations are experimentally observed in time-varying metasurfaces.

## INTRODUCTION

Photonic topological states with unidirectional backscattering-immune property are promising for robust light manipulations (*1*–*7*). These states are typically realized in magneto-optical photonic crystals with broken time-several symmetry, mimicking the quantum Hall effect (*8*, *9*) that was initially discovered in condensed matter physics. However, realizing such states at optical frequencies in static photonic systems remains a challenge mainly because of the weak magneto-optical response of materials at these frequencies. In 2012, Fang *et al.* (*10*) theoretically proposed to realize topologically protected one-way photonic edge states without using the magneto-optical effects in a resonator lattice by harmonically modulating the coupling constants between the resonators in time. In general, anomalous Floquet topological states (*1*, *3*) relying on periodic time modulation can realize unidirectional backscattering-immune propagation though with zero Chern numbers of the bandgaps (*11*). Motivated by the intriguing possibilities to break the time-reversal symmetry via time-periodic modulation and thus to realize one-way photonic edge states in Floquet systems, the photonic Floquet topological insulators (PFTIs) have attracted a growing interest in recent years.

However, up to now, most of PFTIs were realized via static space modulation instead of time modulation. For instance, the first PFTI was realized in an array of evanescently coupled helical waveguides fabricated by femtosecond laser writing method and arranged in a graphene-like honeycomb lattice (*12*). In this setup, the paraxial propagation of light is described by a Schrodinger-like equation, where the propagation direction *z* replaces the role of time and the helicity of the waveguides breaks the *z*-reversal symmetry, thus mimicking the time-periodic modulation and time-reversal symmetry breaking. PFTIs in the coupled helical waveguide arrays have been extensively studied in the past (*13*–*17*). Nonetheless, the three-dimensional (3D) structure of helical waveguide arrays is relatively bulky, making their integration into semiconductor platforms and on chip applications challenging. PFTIs based on static space modulation could also be realized in arrays of coupled microring resonators, where many interesting topological phenomena have been explored and diverse photonic devices and applications have been developed (*18*–*26*). Despite the great progress made in realizing PFTIs via space modulation, these systems are usually made of all-dielectric materials, which do not explicitly break the time-reversal symmetry. As a result, the robustness of their topological protection against large fabrication disorder remains an important practical concern.

Realizing PFTIs via direct time modulation poses considerable challenges, because it requires the implementation of phase-synchronized time-varying modulation signal on large system elements (*27*, *28*). Currently, most proposals on time-varying Floquet topological states are based on numerical simulations (*29*, *30*), except for a few experimental demonstrations. For instance, elastodynamic Floquet topological insulators for acoustic wave have recently been demonstrated in experiment (*28*), which involves the modulation in time the local effective elasticity modulus of a hexagonal array of circular piezoelectric disks. Nagulu *et al.* (*31*) further reported the experiments of the Floquet topological insulator based on quasielectrostatic wave propagation in switched-capacitor networks. Hence, realizing the Floquet topological insulators based on different system platforms and developing previously unknown methods to implement the time modulation protocols not only mark substantial advancements in our ability to accurately manipulate a dynamic system in real time, but these achievements could also offer unprecedented opportunities for practical applications. To date, experimental studies on time-varying Floquet topological insulators in open systems have been exceedingly rare. The extension of the Floquet theory to specific conditions, particularly for time-varying systems in open environments, remains a relatively underexplored area. The mathematical description of the open systems is notably intricate, involving complex nonlinear and nonequilibrium dynamics, as well as the need to account for the environmental interactions. These multifaceted challenges have substantially hindered the exploration of time-varying Floquet phenomena, with experimental realizations being particularly constrained.

In this article, we propose a time-varying metasurface system to explore Floquet topological states theoretically and experimentally in time domain, effectively addressing the challenges in experimental realization of the time-varying Floquet topological insulators in open systems. The metasurface consists of a square array of spoof localized surface-plasmon (SLSP) resonators made of spiral metal patterns printed on a metal-grounded dielectric substrate. The annular arm of each SLSP resonator integrated with PIN diodes is independently programmed using a high-speed field-programmable gate array (FPGA) control system, and, as such, the connections of each resonator to its four nearest neighbors can be independently controlled by the on/off states of the PIN diodes. We first study a periodic driving protocol, where the PIN diodes around one square plaquette are turned on and off sequentially either in clockwise (CW) or counterclockwise (CCW) direction, and demonstrate that the metasurface under this driving protocol exhibits a topological transition as the switching frequency of the PIN diodes increases. In particular, the Floquet harmonic bands are emanated from the fundamental resonance frequency of the SLSP resonators as the switching frequency increases and at specific switching frequencies, anomalous edge states with inherent chirality determined by the handness (CW or CCW) of the switching sequence emerge in the Floquet harmonic bandgaps. Then, electromagnetic (EM) waves are generated at different harmonic orders of the Floquet driving frequency by exploiting the chiral edge states in different Floquet harmonic bandgaps and studying the open-system nature of the time-varying metasurface where the energy can be exchanged with the outside modulation source. In general Floquet systems, energy exchange is closely linked to external sources, as achieving periodic modulation in the system necessarily requires energy input/output; however, these systems typically do not interact with free space directly. In contrast, our metasurface is in direct contact with free space and thus can interact with the free-space EM modes in a nontrivial way. This nontrivial wave interaction precludes a simple tight-binding description of our system, a unique feature that previous Floquet systems have not been able to achieve. Recently, nonlinear topological photonics has been an important research topic in photonics, and the demonstrated topological harmonic waves mediated by the anomalous Floquet edge states in this work provide a route for exploring the nonlinear topological photonic phenomena, because strong nonlinearity of optical materials is not needed. Last, to demonstrate more general time-modulation patterns in our metasurface platform, we propose a large-scale programmable coding scheme to dynamically reconfigure the topology of the propagation paths by controlling the binary states of the meta-unit. The proposed time-varying metasurface not only provides a platform to explore many interesting time-dependent physics (e.g., periodic, quasiperiodic, or even random driving) but also offers promising potentials for applications in metamaterials (*32*–*39*), active integrated devices, and information science due to its real-time reprogrammable capability.

## RESULTS

### Time-varying topological metasurface

**Fig. 1.**
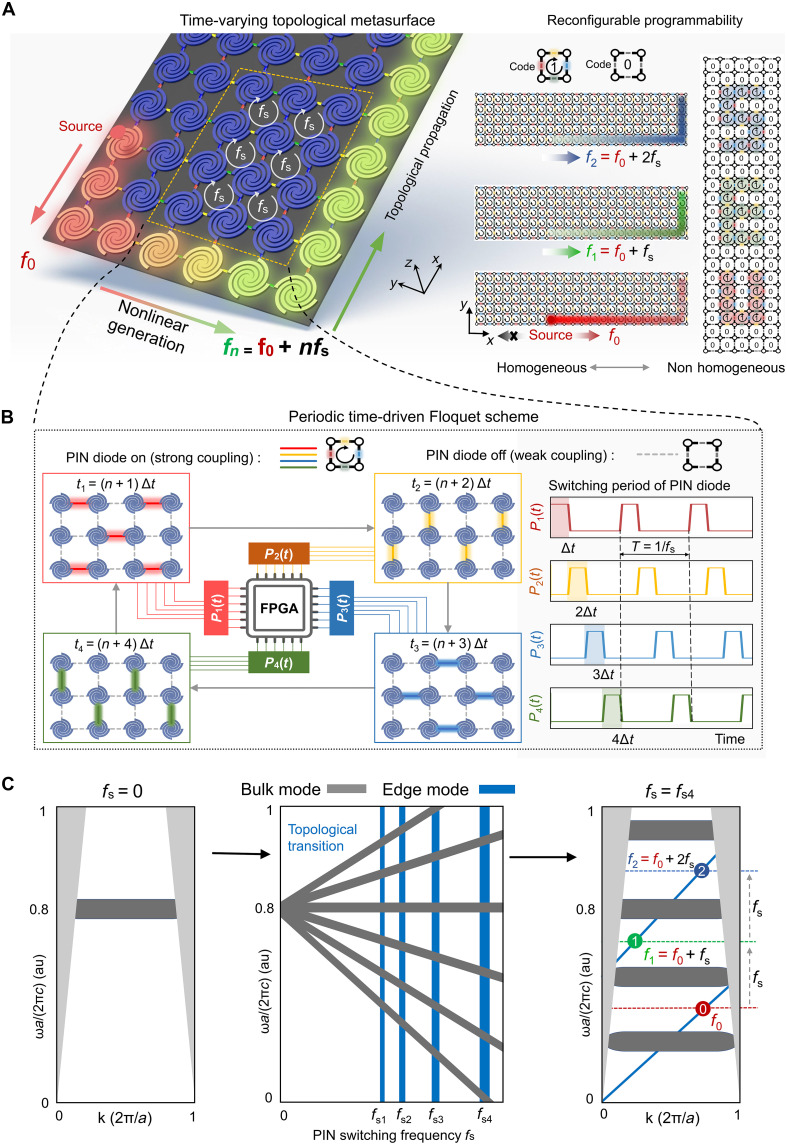
The working principle and main functions of the proposed time-varying topological metasurface. (**A**) The time-varying topological metasurface driven by a four-step switching protocol controlled by FPGA, in which the metasurface is composed of square SLSP resonators, which are represented by the spiral-grooved metal disks on a dielectric substrate. Harmonic generations mediated by the topological edge modes at $f_{0}$, $\;f_{1}$, $f_{2}$ and reprogrammability of the time-varying topological metasurface. For the harmonic generations, a source placed at the edge of the metasurface excites an EM wave propagating unidirectionally along the system edges. Due to harmonic effects, the wave is converted from frequency $f_{0}$ to $f_{1}$ and $f_{2}$ during the propagation (see also the illustration). For the reprogrammability, any coding patterns (“S,” “E,” and “U” as examples here) on the metasurface can be programmed for controlling the topological propagation paths of EM waves. (**B**) The strong/weak couplings of each SLSP resonator to its four nearest neighbors are controlled by the on/off states of the PIN diodes on the connecting arms. During each period *T* of the Floquet driving protocol, the on/off states (highlighted by the colored-solid/uncolored-dashed arms) of the PIN diodes are controlled by the signals $P_{1,2,3,4}\;\left(t\right)$ via FPGA. This four-step driving protocol has an inherent chirality, i.e., the PIN diodes around one square plaquette can be turned on sequentially either in CW or CCW direction. (**C**) The topological transition induced by the PIN switching frequency ( $f_{s}=1\;/\;T$ ), where the Floquet harmonic bands are emanated from the resonance frequencies of the SLSP resonators as the switching frequency increases and at certain specific switching frequencies (labeled by $f_{s1},f_{s2},f_{s3},\text{and}\;f_{s4}$ ), anomalous edge modes will emerge in the Floquet harmonic bandgaps. The right panel shows the Floquet spectrum in the momentum space at $f_{s4}$ with three topological edge modes ( $f_{0},f_{1},\text{and}\;f_{2}$ ) connected by the switching frequency $f_{s}$ . au, arbitrary unit.

The proposed time-varying topological metasurface and its time-periodic driving protocol are shown in Fig. 1A. The metasurface comprises an array of SLSP resonators arranged in a square lattice. Each arm between SLSP resonators contains PIN diodes for programmable connections, which enable diverse harmonic frequency generation and topological propagation. The right part of Fig. 1A schematically shows the harmonic generation and programmable capability of the time-varying topological metasurface. For the harmonic generation, a source with excitation frequency $f_{0}$ placed at the bottom edge of the metasurface can excite an EM wave at the same frequency $f_{0}$ that propagates unidirectionally along the system edges without being scattered by the corners either to the backward direction or to the bulk. However, during the propagation, the intensity of the fundamental wave will decrease and convert to different harmonic waves with frequencies of $f_{n}=f_{0}+nf_{s}\;\left(n=1,2,\ldots \right)$ , where $f_{s}$ is the switching frequency of the Floquet protocol. For the reprogrammable capability of the time-varying metasurface, any coding patterns (for example, “S,” “E,” and “U” in Fig. 1A) based on the same metasurface platform can be programmed by designing and controlling the two digital states “0” and “1” of each unit cell, which could be exploited to dynamically reconfigure the topological propagation paths of EM waves. These unique features of the topological transition, harmonic wave generation, and reconfiguration ability of the time-varying metasurface will be demonstrated below in detail. Each SLSP resonator is composed of a spiral-grooved metal disk on a dielectric substrate and is connected to other SLSP resonators through four identical PIN diodes (colored links in Fig. 1B) situated in between. As such, the connections of the SLSP resonators to their nearest neighbors can be controlled by the on/off states of the connecting PIN diodes. Because the spectral and field confinement properties of the SLSP resonators are crucially dependent on their structural parameters, we conduct a series of numerical calculations to obtain the desired properties by optimizing the structural parameters, such as the length of the curved groove, the number of spokes, and the lattice constant.

While we could, in principle, control the on/off states of the PIN diodes in any prescribed pattern, we prefer a special time-periodic driving protocol that can lead to anomalous Floquet topological edge states without the traditional bulk-edge correspondence, as shown in Fig. 1B. In this protocol, each period T of the switching sequence consists of four steps of equal duration ∆t=T/4 , in which the PIN diodes are switched on and off states on the colored-solid and uncolored-dashed links, respectively. As the SLSP resonant modes are mainly localized around the resonators, the coupling between two nearest neighbor SLSP resonators will be strongly modulated when turning the PIN diodes on and off. One can expect that when the PIN diode is on, the coupling will be strong, and, conversely, when the PIN diode is off, the coupling will be weak. A simple tight-binding model with hopping amplitude varying periodically in time and an intuitive explanation for the emergence of anomalous chiral edge states in this switching protocol are given in section S1. However, the real time-varying metasurface system is beyond the tight-binding description due to the open-system nature and harmonic mode dispersion. Hence, a method based on numerical simulation is developed and used to study the topological properties of the time-varying metasurface. Details on the developed method to simulate time-varying media are shown in section S2. Each PIN diode on the connecting arm can be independently controlled by FPGA according to the voltage modulation signal $P_{1,2,3,4}\left(t\right)$ (for details on the experimental structure design, see section S3). The four-step driving protocol has an inherent chirality due to its capability to execute the switching sequence around a square plaquette in two inequivalent manners (i.e., CW and CCW).

The time-varying metasurface will generate topological transitions when increasing the switching frequency of the PIN diodes, as shown in Fig. 1C. We observe that the edge states, represented by the blue bands, emerge at specific switching frequencies, which we denote as $f_{\text{ss}}$ . Such frequencies have an intrinsic relationship with the SLSP resonance frequency, as detailed in section S13. On the other hand, the bulk modes consist of several Floquet harmonic bands indicated by the dark gray regions. All Floquet harmonic modes are originated from the same resonant mode, with the corresponding frequencies defined as $f_{n}=f_{0}+nf_{s}\;\left(n=1,2,\;\ldots \right)$ . In particular, the horizontal region signifies the fundamental resonant frequency of the SLSP resonators, whereas the rest are harmonics generated thereof, demonstrating the existence of essential harmonic characteristics in the time-varying metasurface. The middle panel of Fig. 1C displays only a limited number of harmonic bands. Theoretically, the number of harmonic bands is infinite. However, as the harmonic order increases, its intensity gradually diminishes. Consequently, the energy levels of higher-order modes are often obscured by the background noise in experiments. The right panel of Fig. 1C illustrates the Floquet edge-state spectrum in the momentum space for illustrating the topological harmonic generation mediated by the edge modes at frequencies $f_{0}$ , $\;f_{1}$ , and $f_{2}$ . The harmonic generation described in our manuscript is not originated from the intrinsic nonlinear susceptibility of the conventional nonlinear optical materials but rather represents an equivalent nonlinearity. This equivalent nonlinearity is induced by the introduction of our periodically driven time-varying metasurface. This type of equivalent nonlinearity can be regarded as an outcome of temporal dimensional synthesis. Moreover, as detailed in (*11*), the “new frequency” discussed in the paper actually corresponds to a quasienergy distribution within the Floquet band structure induced by periodic driving rather than arising from the traditional nonlinear optical process.

### Topological transition of the time-varying metasurface

**Fig. 2.**
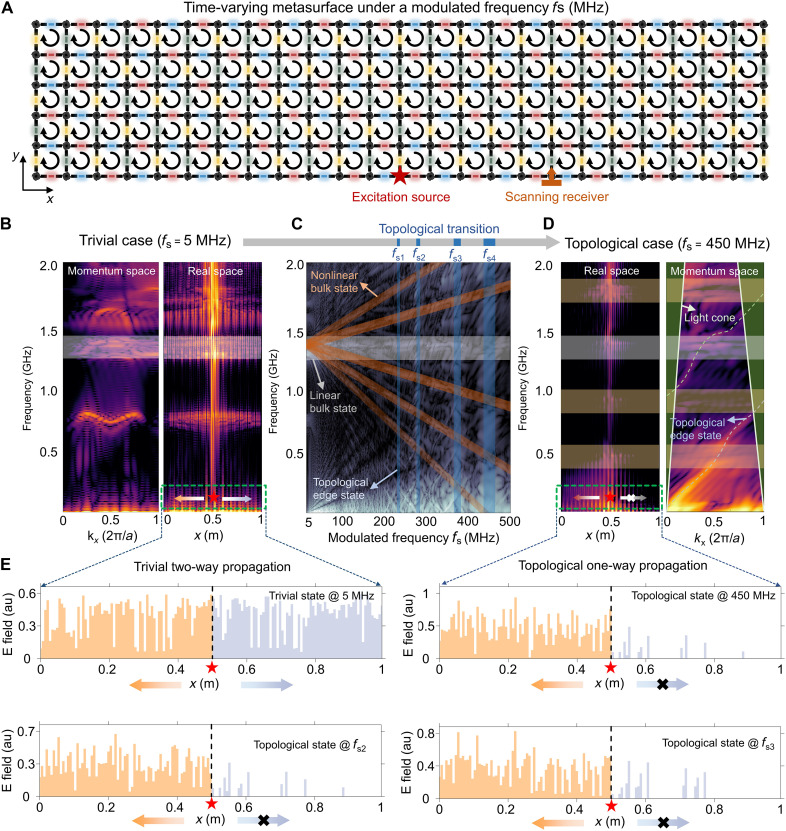
Simulation and experimental results of the topological transitions in the time-varying metasurface induced by PIN switching. (**A**) Schematic illustration of the time-varying metasurface under a CW four-step sequential driving protocol, where the source for exciting the topological edge modes and the receiver for scanning the edge mode profiles are marked. (**B**) Trivial quasienergy spectra of the metasurface in real (right) and momentum (left) spaces when $f_{s}=5$ MHz, where the frequency windows around the fundamental frequency (1.35 GHz) of the SLSP resonators are marked by the bright regions. The spectra in both real and momentum spaces exhibit almost symmetrical characteristics, where no edge states exist apart from the bulk states. (**C**) Topological transition of the metasurface when increasing the switching frequency $f_{s}$ , where a series of harmonic bands (brown regions) are measured in experiments (section S3) and edge states (blue regions) emerge between the harmonic bands at specific switching frequencies of $f_{s1}=225\;$ MHz, $f_{s2}=275\;$ MHz, $f_{s3}=350\;$ MHz, and $f_{s4}=450\;$ MHz. (**D**) Topological quasienergy spectra of the metasurface in real (left) and momentum (right) spaces when $f_{s}=450\;$ MHz, where the edge states (denoted by the white dashed lines) emerge in the momentum space apart from the four equally spaced bulk bands (marked by the bright regions). The observed positive group velocity of the edge states in the momentum space correlates well with the fact that EM waves only propagate to the right direction in the real space. (**E**) Electric field distributions of trivial two-way propagation at $f_{s}=5$ MHz and topological one-way propagation (to the left) at $f_{s4}=450\;\text{MHz},f_{s2}=275\;$ MHz, and $f_{s3}=350\;$ MHz.

In this section, we show that the proposed time-varying metasurface will exhibit topological transitions as the PIN diode switching frequency increases via the proposed numerical calculations (for details, see Materials and Methods). The metasurface used in calculations is made up of 25 by 6 SLSP resonators. In 2012, a structured metallic cylinder was proposed to support the SLSP modes in the low-frequency range (*40*). The SLSP resonators considered here are based on the spiral-wave metal disk with higher field enhancement (*41*). This design has some appealing features (such as ultrathin), and the spirals with long bending grooves could result in a much smaller particle size than the wavelength of the resonant mode, enabling the SLSP resonator to manipulate the subwavelength-scale fields. Each SLSP resonator is connected to four identical neighboring resonators via switchable PIN diodes situated in between (see the schematic in Fig. 2A). In our numerical calculations, the SLSP resonators have a radius of 20 mm and thickness of 2 mm and are made of metallic conductors. The PIN diodes are modeled as a time-varying material, in which different conductivities are used to characterize the switching states.

To study the topological property of the time-varying metasurface when increasing the PIN diode switching frequency, we applied a 0- to 2-GHz broadband Gaussian pulse as the excitation source signal to the metasurface and recorded the spectra of the received signal at different locations as the PIN diode switching frequency is gradually increased from 0 (static) to 500 MHz (see the schematic in Fig. 2A). We remark that the spectrum shown in Fig. 2C reveals detectable responses at two fundamental frequencies: One is around zero frequency, which is unsuitable for exploring the topological property of the metasurface due to its low frequency and weak response, while the other is around 1.35 GHz, which supports a more notable response and will be focused in the analysis. The spectrum shown in Fig. 2C exhibits a prominent fundamental resonance at 1.35 GHz, at which the system response is particularly notable; therefore, this frequency will be the primary focus of our subsequent analysis. Here, we denote this resonance frequency as $f_{r}$.

In Fig. 2C, we observe prominent bulk Floquet harmonic bands (*42**,* *43*), which are radiated out around this frequency by forming a fan-shaped structure when the PIN diode switching frequency increases. These bands correspond to the fundamental frequency $f_{0}$ (the horizontal one) and their harmonics appearing at $f_{0}\pm nf_{s}$ , where n is the harmonic order and $f_{s}$ is the switching frequency of the PIN diodes. The measured responses (highlighted by the brown regions) show good agreement with the simulation results and the analysis based on the harmonic generation. The responses at higher frequencies in general are much stronger than those at lower frequencies. Apart from the bulk states, anomalous edge states at specific switching frequencies ( $f_{s0}=180\;$ MHz, $f_{s1}=225\;$ MHz, $f_{s2}=275\;$ MHz, $f_{s3}=350\;$ MHz, and $f_{s4}=450\;$ MHz) are also observed in the spectrum, which are marked by the blue regions. These specific switching frequencies are primarily determined by the resonance frequency of SLSP, which are expressed approximately as (*44*) $f_{\text{ss}}=\frac{f_{r}}{k}\;\left(k=1,2,3,\;\ldots \right)$ . The observed topological transitions at specific switching frequencies of 180, 225, 275, 350, and 450 MHz correspond to k=7,6,5,4,and3, respectively. Theoretically, stronger chiral edge state responses should be observable at specific switching frequencies of 675 and 1350 MHz; however, due to experimental limitations, we were only able to observe the specific switching frequency corresponding to k=3 . To maintain consistency with the experimental results, we have chosen not to present observations at higher frequencies. On the other hand, when k exceeds 7, similar phenomena become increasingly difficult to observe due to the weak responses of the system. Further analysis can be found in section S13. The size of the topological bandgaps (indicated by the width of the blue regions) is larger at high switching frequency due to the fan-shaped spectral structure, indicating that the edge states at higher switching frequencies are more localized around the metasurface edges. The results in Fig. 2C show that the edge states can appear or disappear by emerging from or converting into the bulk states when varying the switching frequency. At the frequency with emergent edge states, multiple topological bandgaps between different harmonic bands can be clearly identified.

To better contrast the topological transition in Fig. 2C, we present the spectral responses in both real and momentum spaces in two cases: low switching frequency at 5 MHz (see Fig. 2B) and high switching frequency at 450 MHz (see Fig. 2D). When the PIN diode switching frequency is 5 MHz, the system is approximately static. To study the response of metasurface at this frequency, we placed the excitation source at the middle along the system edge, such that the responses at both sides of the excitation source could be faithfully detected. The real space spectrum of the metasurface covering the entire range of metasurface is shown in the right panel of Fig. 2B. Clearly, the spectrum is almost symmetric along the forward and backward directions with respect to the source location and does not have clear signature of one-way propagation. The bulk states around the fundamental resonance frequency of SLSP resonators are marked by the bright region. By performing Fourier transform of the real space spectrum, the response in the momentum space is obtained, which is presented in the left panel of Fig. 2B. From the results, we observe that no edge sates exist in the spectrum apart from the bulk states.

To show the emergence of one-way edge states at higher switching frequencies, the response of metasurface at 450 MHz is presented in Fig. 2D, in which the edge states are most pronounced, facilitating the analysis of their topological properties. In the real-space spectrum (see the left panel), one-way edge states propagating to the left side of the excitation source are clearly observed. The Fourier-transformed spectrum in the momentum space is shown in the right panel, from which one can see the edge states marked by the white dashed lines. The chiral edge states at the higher harmonics are not as obvious as those at the fundamental frequencies. This is primarily attributed to the fact that the chiral edge states at higher harmonics are more likely to radiate out of the time-varying metasurface, as they are closer to the light cone. More specifically, the chiral edge states located above the light cone can radiate out of the time-varying metasurface, forming leakage modes. In contrast, the chiral edge states below the light cone remain confined in the time-varying metasurface, with their energy not leaking outside. Consequently, these states can propagate with the minimal radiation loss on the time-varying metasurface. Therefore, the fundamental resonant frequency and lower-order chiral edge states typically exhibit stronger energy, whereas the energy of higher-order harmonics is progressively diminished. The group velocity of the edge states (i.e., the slope of the edge state dispersion curve) is positive, indicating that the edge states propagate only to the right side of the source, which agrees with the real-space spectrum. As the edge states with only one propagation direction exist in the spectrum, they cannot be backscattered as long as the bandgaps protecting these states persist.

The inherent chirality of edge states is due to the CW nature of the switching sequence of PIN diodes around one plaquette. In practice, one can change the switching sequence between CW and CCW directions, thus effectively controlling the propagation direction of the edge states. The electric field distributions of the trivial two-way propagation at $f_{s}=5$ MHz and the topological one-way propagation (to the left) at $f_{s2}=275\;$ MHz, $f_{s3}=350\;$ MHz, and $f_{s4}=450\;\text{MHz}\;$ are further presented in Fig. 2E, which clearly show the contrast between the trivial two-way and topological one-way propagation. The numerical results presented here unambiguously demonstrate that the topological transitions and anomalous edge states can be induced in the proposed metasurface via the four-step chiral switching protocol, which will lay a solid foundation on their practical applications. Here, we should clarify that the generation mechanism of topological edge states in our system is not merely a trivial harmonic generation or traditional frequency conversion process. As illustrated in fig. S13, in the initial system without time modulation, only one intrinsic bulk-state region (depicted by black region) is present, and there are not any topological edge states. When a traditional time-varying scheme is used, several harmonic bulk-state regions are generated, leading to a frequency conversion phenomenon. However, the topological edge states cannot be generated in this traditional time-varying scheme. Therefore, the harmonic bulk states can be generated in any time-varying systems, but the topological edge states cannot be guaranteed to exist in the wave systems that are time modulated. In contrast, within our time-varying scheme, the topological edge states (depicted by red line) can be observed between adjacent bulk-state regions at specific switching frequencies. A more comprehensive theoretical explanation of our time-varying scheme has been detailed in section S1. These results indicate that the harmonic bulk states can be generally generated in any time-varying wave systems, whereas the Floquet topological states can only be generated in a specific time-varying scheme.

### Harmonic Floquet topological states

**Fig. 3.**
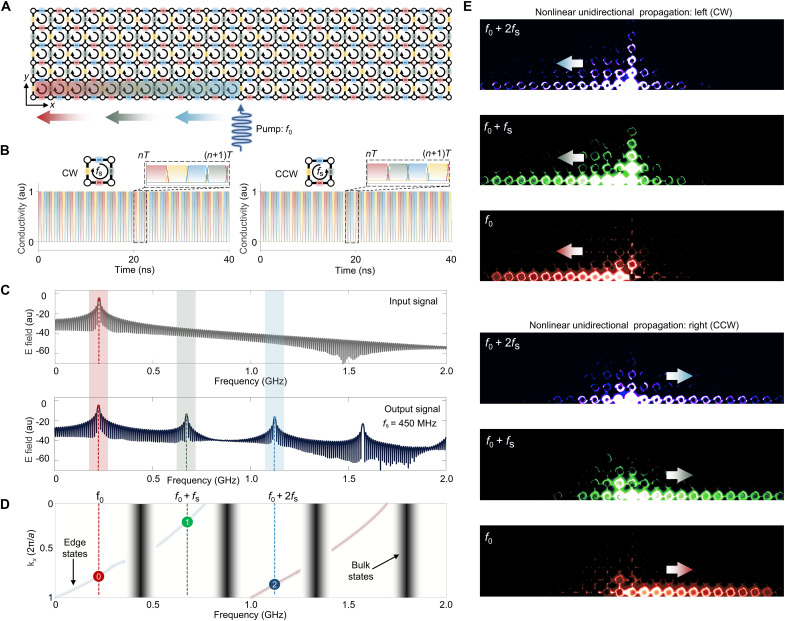
Harmonic wave generation and unidirectional propagation in the time-varying metasurface. (**A**) The schematic of one-way harmonic wave generation with the pump frequency $f_{0}$ in the time-varying metasurface under the CW driving. (**B**) The time-series signals for achieving the CW and CCW driving protocols. (**C**) The electric field response (output signal) in the frequency domain of the metasurface under pump (input signal) frequency $f_{0}$ and switching frequency $f_{s}=450$ MHz. Clear response peaks at $f_{0}+nf_{s}$ (*n* = 1, 2, and 3) in the output signal are observed. (**D**) Floquet bulk and edge state spectra in the momentum space, where the edge states corresponding to the response peaks in (C) are marked. (**E**) Electric field distributions ( $|\;E_{z}\;|$ ) of the generated harmonic waves and their unidirectional propagation under CW (top three panels) and CCW (bottom three panels) driving. The pump frequency is at $f_{0}=200\;\text{MHz},$ whereas the generated harmonic waves are at $f_{0}+f_{s}\;$ and $f_{0}+2f_{s}\;$ with the driving frequency of $f_{s}=450\;\text{MHz}$.

An important feature of the time-varying metasurface is its capability to generate harmonic waves via the topological edge states in different bandgaps. An illustration of this phenomenon is schematically shown in Fig. 3A. To study the harmonic wave generation, we applied an external source with pump frequency of $f_{0}=200\;$ MHz to the time-varying metasurface at the middle of bottom edge. The field distribution of the pump source in frequency domain is shown in the top panel of Fig. 3C, and the response of metasurface under the driving frequency of $f_{s}=450$ MHz is presented in the bottom panel. From the field distribution, we observe additional peaks located at different harmonic orders of the driving frequency $f_{s}$ , apart from the main peak at the pump frequency $f_{0}=200\;$ MHz. The frequency conversion efficiencies at the first, second, and third harmonic frequencies are 4.17, 1.48, and 0.15%, respectively. The mechanism of Floquet replicas of the edge state modes in different bandgaps ensures a high level of phase matching for the harmonic generation in our time-varying topological metasurface platform (for details, see section S14). Furthermore, we explored the mathematical mechanisms underlying the harmonic generation in time-varying metasurfaces (detailed derivations are given in section S10). Based on Maxwell’s equations, we derived the spectral components generated by introducing the pump frequency, with the expression for the surface-wave electric field $E_{s}\left(f\right)$ given as

$$\begin{array}{c} \begin{array}{c} E_{s}\left(f\right)=\sum_{n=-\infty }^{\infty }\frac{\left(i+1\right)\left[2\text{sin}\left(\frac{n\pi }{2}+\frac{\pi f_{c}T}{2}\right)+1-\text{sin}\left(n\pi +\pi f_{c}T\right)-\text{cos}\left(n\pi +\pi f_{c}T\right)\right]}{i\left(nf_{0}+f_{c}\right)}\\ E_{i}\left(f-f_{c}-nf_{0}\right) \end{array} \end{array}$$ (1)

where n denotes the harmonic order, $f_{0}=\frac{1}{T}$ is the modulation frequency, $f_{c}$ is the frequency of the monochromatic input signal, and the incident wave is defined as $E_{i}\left(f\right)$ . The quasienergy spectrum of the response signal in the momentum space is given in Fig. 3D, in which the response peaks correspond to the topological edge states at frequencies of $f_{0}+nf_{s}\;\left(n=1,2,\;\ldots \right)$ . Here, we should note that not all harmonic generations of bulk states can result in the chiral edge states, although the generation of chiral edge states is dependent on the harmonic generation of bulk states. Our theoretical and experimental results show that the chiral edge states only emerge in each gap between adjacent harmonic generations of bulk states under some specific time-varying frequencies and schemes. If the edge states within different bandgaps have negative group velocities, the generated harmonic waves will unidirectionally propagate to the right direction along the bottom edge of the metasurface.

The electric field distributions of the generated harmonic waves in the real space are illustrated in Fig. 3E, which propagate unidirectionally to the right direction of source along the bottom edge, agreeing with their positive group velocities in Fig. 3D. This confirms that all waves participating in the harmonic generation are edge states, since the fields do not penetrate to the bulk region but decay exponentially away from the edge. We highlight that there are no external sources at the first and second harmonic frequencies, and the generation of waves at these frequencies is purely due to the open-system nature of metasurface, where energy can be injected to the metasurface system through time-varying modulation. This is very different from the conventional way of nonlinear wave generation in static systems via the intrinsic nonlinear susceptibility of conventional nonlinear optical materials. As a result, our equivalent frequency conversion efficiency is substantially higher than that of the conventional nonlinear optical materials. As the first and second harmonic frequencies of the edge states are both located in the topological bandgaps (see Fig. 3D), they enjoy the same topological protection as the fundamental frequency, i.e., the backscattering-immune one-way propagation.

As the propagation direction of the waves is locked to the chirality of driving protocols (CW and CCW switching sequences), changing the direction of switching sequence could lead to the harmonic-wave generation to the left side of source, which is confirmed in the numerical calculations (see the top three panel of Fig. 3E). The time-series signals for achieving the CW and CCW driving protocols are shown in Fig. 3B. As the two switching sequences are time-reversal partners, changing the switching of the sequence from CCW to CW will lead to the edge states that are mirror symmetric along the frequency axis to the ones presented in Fig. 3D. Consequently, the slope of the edge states (group velocity) will be reversed to negative, leading to unidirectional propagation to the left side. It should be noted that the spectral components produced by introducing the pump frequency will vary, requiring the electric-field expression for the surface wave $E_{s}\left(f\right)$ to be reformulated as

$$\begin{array}{c} \begin{array}{c} E_{s}\left(f\right)=\sum_{n=-\infty }^{\infty }\frac{\left(i+1\right)\left[2\text{sin}\left(\frac{n\pi }{2}+\frac{\pi f_{c}T}{2}\right)-1-\text{sin}\left(n\pi +\pi f_{c}T\right)+\text{cos}\left(n\pi +\pi f_{c}T\right)\right]}{i\left(nf_{0}+f_{c}\right)}\\ E_{i}\left(f-f_{c}-nf_{0}\right) \end{array} \end{array}$$ (2)

The harmonic-wave generation mediated by the edge states in different bandgaps offers a mechanism to study nonlinear phenomena in the topological photonic systems with promising potentials in practical applications. To further illustrate the programmability of the proposed time-varying metasurface, we investigate a large-scale metasurface with inhomogeneous modulation patterns. This enables the realization of near-field distributions corresponding to letter shapes (“S,” “E,” and “U”) individually, as detailed in section S9.

### Experimental verification

**Fig. 4.**
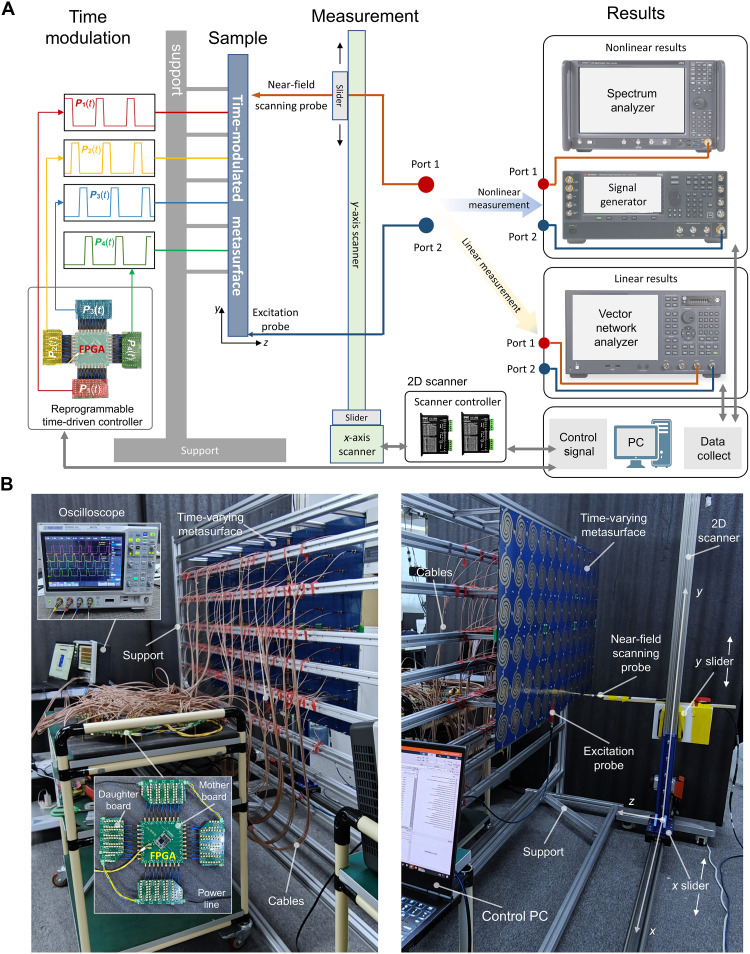
The near-field measurement system and FPGA-based control module for the time-varying metasurface. (**A**) The schematic of near-field measurement platform, where the probe is scanning in the *xy* plane, whereas the 2D scanner is placed directly in front of the metasurface to test its surface electric fields. The metasurface is positioned in the *xy* plane perpendicular to the ground, and FPGA and its control cables are set behind the metasurface for time modulations. The two port of the scanner can selectively connect two test systems respectively for linear (VNA) and harmonic testing (spectrum analyzer and signal generator), respectively. In the test system, PC is used as the control terminal to complete the test data collection, 2D scanner, and FPGA control. (**B**) Experimental scenario of near-field measurement. Behind the metasurface is the FPGA-based high-speed signal control module, consisting of a daughter board and a mother board. The mother board generates time-varying control signals from FPGA and sends them to the daughter board. The driver array of the daughter board helps achieve stable signal output and monitor the signal waveform. An oscilloscope is connected to the output end of the daughter board through coaxial cables. The 2D scanner in front of the metasurface measures the near-field electric field distribution.

To verify the topological properties of the time-varying metasurface, we fabricate an experimental sample of metasurface and investigate its responses to external excitation. The near-field measurement system and FPGA-based control module for executing the switching protocol of the PIN diodes are shown in Fig. 4, and more details on the structure design and experiments are presented in section S4. As shown in Fig. 4A, the metasurface fixed on a support is tested via two ports: an excitation probe and a scanning probe, which can be selectively connected to the linear and harmonic measurement systems. The linear measurement is performed by vector network analyzer (VNA) under the same frequency excitation, while the harmonic measurement is for the spectral harmonic responses by the single-tone source. A personal computer (PC) is used as the control terminal to read instrument measurement data, control the scanner movement, and switch the FPGA signal. The four quarter-cycle signals generated by FPGA are connected to the corresponding PIN diodes through coaxial lines of equal length.

The time modulations are achieved by applying the signals on each SLSP arm through the radio frequency (RF) cables connected behind the metasurface, and the detailed time-modulation control system is shown in Fig. 4B. Specifically, the high-speed phase synchronization signal is generated by the FPGA core board, which is first sent to the four daughter boards (high-speed register array) through equal-length coaxial lines. The register array then drives all diodes to achieve the high-speed synchronization time-varying control and is monitored by an oscilloscope. The time delay remains in the picosecond range even in the presence of fabrication errors, because of the nearly equal length of each RF cable. For instance, in the experiments conducted at a switching frequency of 180 MHz, the time delay contribution to the activation period of each PIN diode is less than 0.72%, significantly lower than the threshold for system time error, thereby ensuring the robustness required for system operation. Furthermore, we provide a more detailed analysis of the effects of time desynchronization and time jitter on system robustness in section S12, which validates the robust propagation characteristics of our system. The configuration of the experimental platform is given in Materials and Methods and section S5. The 2D scanner in front of the metasurface measures the near fields by moving the coupled probe (fixed on *y* slider) in the *xy* plane. The excitation probe is soldered to the center of the bottom unit and connected to the instrument via a coaxial line.

**Fig. 5.**
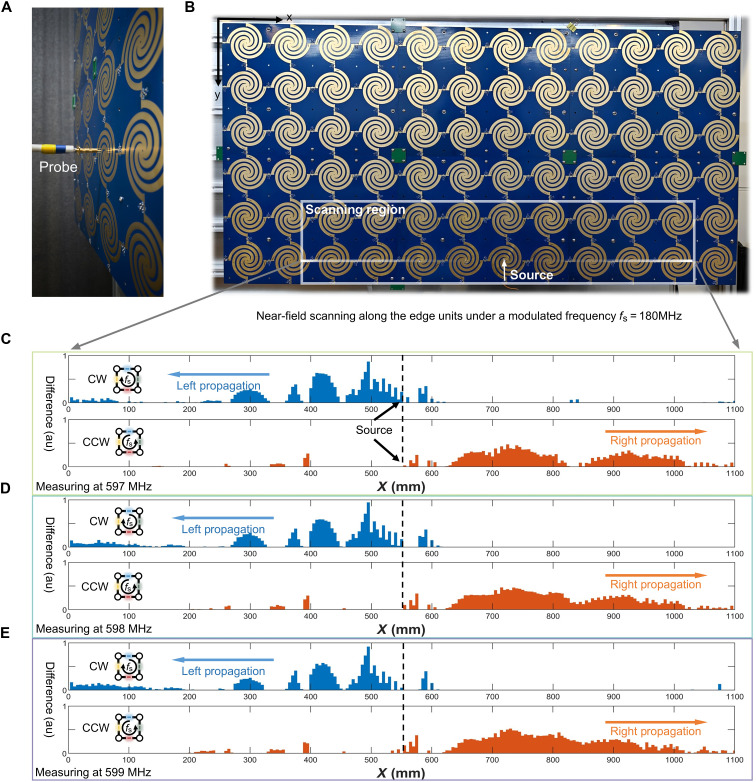
Experimental verification of the unidirectional edge propagation via near-field scanning measurement. (**A**) The illustration of near-field measurements using a scanning probe. (**B**) Experimental sample of the time-varying metasurface, where the white rectangular box marks the near-field scanning region and the white line within the scanning region indicates the scanning path for characterizing the unidirectional edge propagation. The excitation source is loaded by a coaxial probe on the metal arm of the central SLSP resonator. (**C** to **E**) The measured electric-field amplitude difference between the CW and CCW driving protocols along the white line marked in (B). The switching frequency is 180 MHz, while the measuring frequencies are 597, 598, and 599 MHz in (C) to (E), respectively.

The unidirectional propagation of the topological edge states under time modulation is studied in a metasurface consisting of 6-by-12 SLSP resonators, which is produced by the printed circuit board (PCB) technology and is composed of multilayer dielectric plates. The metasurface is spliced by six sub-boards, which is vertically fixed on the metal bracket, as shown in Fig. 5B, and the connections of the edge units between two submetasurfaces are directly soldered (fig. S6). Specific structural settings, such as diode loading and bias circuits, are shown in fig. S6. To realize the periodic control waveform of the high-frequency signal, the control signal (section S6) is given by FPGA and drives each group of PIN diodes through the register array. The experimental control waveform results are given in fig. S8.

By switching the time-varying driving direction of the diode (the CW and CCW directions shown in Fig. 5), the topological edge state propagation can be generated in the rightward and leftward directions. The experimental results are given in Fig. 5 (C to E), which are measured at 597, 598, and 599 MHz when the switching sequence of the PIN diodes is CW and CCW, respectively. In our experiments, we selected a specific switching frequency of 180 MHz, which maintains consistency with the previously mentioned relationship between specific switching frequency and resonance frequency. To display the experimental results on unidirectional propagation more clearly, we present the normalized difference between the electric field amplitudes along the two propagation directions, from which the unidirectional propagation properties under the CW and CCW switching sequences can be more clearly observed. We notice that the energy difference between the rightward and leftward unidirectional propagation in the near-field experiments is not as pronounced as that in simulation results, mainly for the following reasons: (i) The ideal time-varying control (periodic square wave) in calculations can be loaded accurately to the unit connections without any influence of the bias circuit, whereas in experiments, the FPGA control waveform is not ideal, which is also subjected to irrational impedance matching caused by the bias circuit. (ii) The isolation of the PIN diodes and nonideal components in the bias circuit lead to nonideal filter circuits, which, in turn, affect the harmonic side state effect. (iii) The stability of the control source is limited. The high control signal of FPGA has a deviation of about 1 MHz, which will affect the harmonic side states. Nonetheless, the experimental results shown in Fig. 5 (C to E) show clear signatures of the unidirectional propagation under the CW and CCW driving sequences.

## DISCUSSION

We proposed a time-varying metasurface platform to explore the topological physics, harmonic-mode interaction, and reprogrammable functions. The metasurface consists of the SLSP resonators arranged in square lattice, where the connections of each resonator to its four nearest neighbors can be independently controlled by the on/off states of PIN diodes. We first explored a Floquet driving protocol where the four PIN diodes around one plaquette are turned on and off sequentially either in CW or CCW manner. We demonstrated that the metasurface under this driving protocol exhibits a topological transition as the switching frequency of the PIN diodes increases. In particular, the Floquet harmonic bands are emanated from the fundamental resonance frequency of the SLSP resonators; as the switching frequency increases and at specific switching frequencies, anomalous edge states emerge within the gaps between the Floquet harmonic bands. These edge states feature inherent chirality, which is determined by the handness of the switching sequence. As the edge states exist within different Floquet harmonic bandgaps, harmonic-wave generation mediated by these edge states was successfully demonstrated. The mechanism to generate the harmonic waves in our time-varying metasurface system is very different from the conventional method relying on the material’s nonlinearity. While the materials of our system do not have any nonlinearity, the time modulation makes the metasurface an open system, which can exchange energy with the outside modulation source, thus enabling the generation of the harmonic waves at different harmonic orders of the Floquet driving frequency. Experiments were performed to generate the Floquet harmonic bands and show the unidirectional propagation of the anomalous edge states.

To demonstrate more general time-modulation patterns to be encoded in the proposed metasurface system, we explored an inhomogeneous coding pattern, where on-demand reconfiguration of the topological propagation paths was successfully achieved. This time-varying metasurface system represents a substantial breakthrough in the experimental demonstration of time-varying Floquet topological insulators in open systems, not only providing a novel platform to explore interesting physical phenomena but also offering promising prospects for various application scenarios due to its powerful reconfigurability. Specifically, this system serves as an ideal experimental platform for studying the cutting-edge physical issues such as photonic time crystals, time-reversal symmetry breaking, and topological phase transitions in the time dimension. Because of its high reconfigurability, we can flexibly adjust the time-modulation parameters according to different physical needs, enabling precise control and observation of various time-related physical phenomena. The flexibility and controllability offer the possibility to explore novel spatiotemporal topological structures beyond the traditional spatial topology, potentially leading to the discovery of unreported topological phases and phase transition laws in the time dimension. From an application perspective, this time-varying metasurface system has broad prospects in ultrafast optical devices, reconfigurable photonic chips, and information storage and processing in the time dimension, laying an important foundation for the development of next-generation photonic devices based on the time modulation.

## MATERIALS AND METHODS

### Numerical calculations of the time-varying metasurface

All numerical computations were carried out using the proposed equivalent modeling of time-varying media (as detailed in section S8) in combination with the corresponding numerical algorithms (described in section S2). Excitation ports were defined as discrete ports. The near-field results of the metasurface were obtained at a distance of *z* = 1 mm above the upper surface of the metal layer. For the equivalent modeling of the PIN diode, the conductivity of the equivalent time-varying medium model was implemented on the connecting arms, with values of $5.8\times 10^{7}$ and 0S/m assigned to represent the on and off states of the PIN diode switch, respectively. These conductivity values were determined through curve fitting of the current-voltage curve (namely, the *I*-*V* characteristic) of the realistic PIN diode used in the study. By dynamically adjusting the conductivity parameters in the time-domain simulations, we simulated a realistic time-varying metasurface. To obtain the band structure of the time-varying metasurface, we computed the near-field distributions at the same cross section for various frequencies using the modified TDFIT numerical algorithm (*45*–*53*). Subsequently, using spatial Fourier transform (see Eq. 3) to convert the EM field from real space to wave vector space, we evaluate the band structures for different switching frequencies. In the wave vector space, the relationship between the wave vector **k** and the frequency ω can be established, thereby representing the band structure of the time-varying metasurface (see fig. S11 for more details).

F(kx,ky)=∫yminymax∫xminxmaxE(x,y)e−i(kxx+kyy)dxdy (3)

where a is the lattice periodicity. $y_{\text{min}}$ and $y_{\text{max}}$ are the minimum and maximum values of the position along the *y* axis of the topological metasurface, respectively. $x_{\text{min}}$ and $x_{\text{max}}$ are the minimum and maximum values of the position along the *x* axis of the topological metasurface, respectively. E(x,y) is the electric field distribution at the cross section where *z* = 1 mm above the metasurface.

### Experimental measurements and configurations

The metasurface sample is produced via the PCB technology and soldered by surface-mount technology. The F4B substrate (ε_r_ = 2.65, tan δ = 0.003) is used with the thickness of 3 mm. The detailed installation is given in fig. S6. The near-field measurement is performed using a 2D scanner, as illustrated in Fig. 4. The time-varying switching of the PIN diodes requires high-speed and synchronous periodic voltage control signals, which are generated by an FPGA core board (AMD Zynq-7020). The six submetasurfaces have a total of 288 time-varying switching channels. Each connecting path requires 2 PIN diodes (MADP-000907-14020), driven by a shift register chip. To make the high-frequency signal waveform not be distorted, it is necessary to ensure impedance matching in the control circuit. Therefore, the complete path of the control signal uses 50-ohm impedance matching (coaxial line or microstrip line). To guarantee the temporal synchronization of control signals arriving at the diodes, all coaxial lines and feedline lengths on the control board are kept equal. Even in the presence of time discrepancies, the contribution of time error during each PIN diode activation period remains below 0.72%, significantly lower than the threshold for system time error, thereby ensuring the robustness required for system operation. Because the four time-varying periodic control signals are sequentially delayed by ¼ cycle (Fig. 1B), the drive circuit is composed of four sets of shift-register arrays, which are injected with drive signals from the FPGA. A two-port VNA (Keysight N5063) is applied for the linear measurement, while spectrum analyzer (Keysight N9040) and signal generator (Keysight E8257D) are used for the harmonic measurement.

## Supplementary Materials

### This PDF file includes:

Supplementary Text Figs. S1 to S18 References
