# Supplementary material for: Floquet topological states in time-varying metasurfaces
Source: Sci Adv. 2025 Sep 26;11(39):eadx9025. doi: 10.1126/sciadv.adx9025 (PMC12466921; doi:10.1126/sciadv.adx9025)
Supplement: Supplementary file 1 — Supplementary Text Figs. S1 to S18 References [file sciadv.adx9025_sm.pdf]

Science Advances
AAAS

Supplementary Materials for

Floquet topological states in time-varying metasurfaces

Qian Ma *et al.*

Corresponding author: Jian Wei You, jvyou@seu.edu.cn; Tie Jun Cui, tjcui@seu.edu.cn

Sci. Adv. **11**, eadx9025 (2025)
DOI: 10.1126/sciadv.adx9025

This PDF file includes:

Supplementary Text
Figs. S1 to S18
References

Section 1: Tight-binding description of the time-varying metasurface

In this part, we provide a simple tight-binding description of the time-varying metasurface studied in the main text. Note that due to the harmonic effects of the resonant modes and the open system nature of the time-varying metasurface, a complete characterization of its physics is beyond the tight-binding model presented here. Nonetheless, the tight-binding model can offer some intuitive insights about the topological properties of the metasurface. To construct the tight-binding model, we take the resonant mode of the spoof plasmonic resonator as a particle hopping on a square lattice. Due to the specific features of the four driving steps, the square lattice can be considered as bipartite (see the white and black sites in Fig. S1a). Moreover, the on and off status of the PIN diodes can be modeled by the presence and absence of nearest-neighbor hopping, respectively. Each driving period with time T consists of four steps (each with duration of T/4), during which the particle can only hop along the connected nearest-neighbor lattice sites (see Fig. S1a).

The tight binding model for the four-step driving protocol shown in Fig. S1a can be described by the following Hamiltonian in each period T, (*11*)

$$H = \begin{cases} J_{\mathrm{red}} \sum_{<ij>} a_j^{\dagger} a_i\,, & 0 < t < T/4 \\ J_{\mathrm{brown}} \sum_{<ij>} a_j^{\dagger} a_i\,, & T/4 < t < 2T/4 \\ J_{\mathrm{blue}} \sum_{<ij>} a_j^{\dagger} a_i\,, & 2T/4 < t < 3T/4 \\ J_{\mathrm{green}} \sum_{<ij>} a_j^{\dagger} a_i\,, & 3T/4 < t < T \end{cases} \qquad (1.1)$$

where a_i and $a_i^{\dagger}$ are the annihilation and creation operators for a particle on lattice site i; and in each step with duration of T/4, hopping can only happen between the nearest-neighbour lattice sites that are connected by the corresponding solid lines in Fig.S1(a). After applying the Fourier transform of $a_i = \sum_k e^{ik.r_i} c_k$ and $a_i^{\dagger} = \sum_k e^{-ik.r_i} c_k^{\dagger}$, the Hamiltonian (1.1) in momentum space is written as

$$H(t) = \sum_k \begin{pmatrix} c_{kA}^{\dagger} & c_{kB}^{\dagger} \end{pmatrix} \begin{bmatrix} 0 & H_{12}(k_x, k_y, t) \\ H_{21}(k_x, k_y, t) & 0 \end{bmatrix} \begin{pmatrix} c_{kA} \\ c_{kB} \end{pmatrix} \qquad (1.2)$$

where A and B refer to the white and black sublattice sites and $H_{12}(k_x, k_y, t) = H_{21}^{*}(k_x, k_y, t) = J_1(t) e^{ik_x} + J_2(t) e^{ik_y} + J_3(t) e^{-ik_x} + J_4(t) e^{-ik_y}$ with $J_i(t) = J,$ for $(i-1)T/4 < t < jT/4,$ and $J_i(t) = 0,$ otherwise. Note that we assume the hopping strengths within each

step are equal, i.e., $J_{\text{red}} = J_{\text{brown}} = J_{\text{blue}} = J_{\text{green}} = J$.

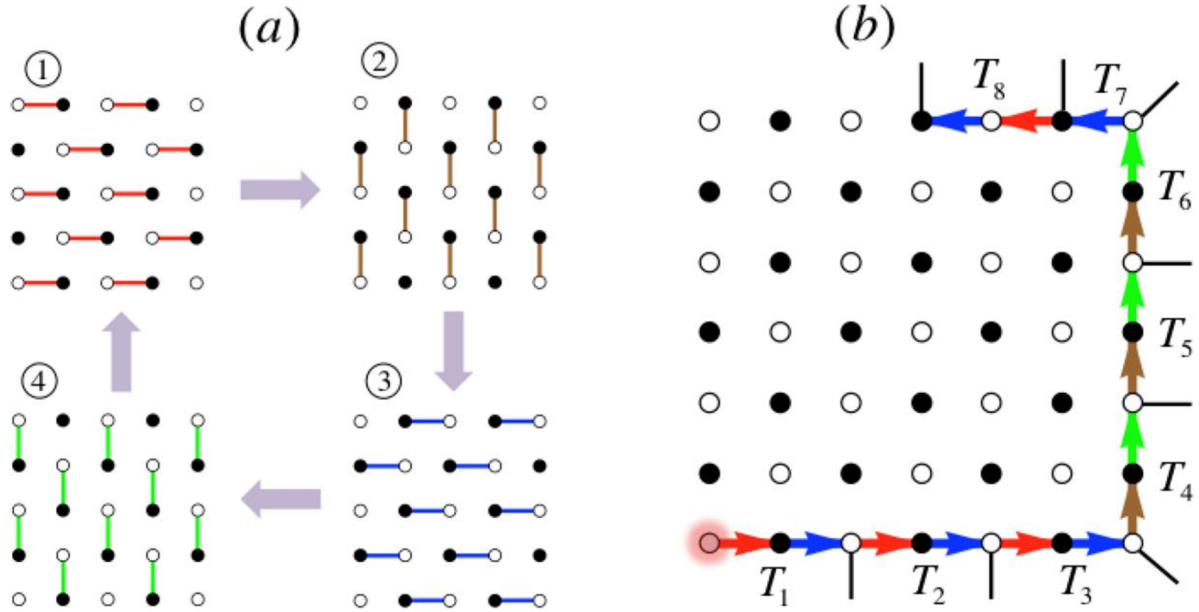

Fig. S1. Driving protocol of the time-varying metasurface. a, Driving protocol of the time-varying metasurface considered in the main text modelled by a simple tight-binding description on a bipartite square lattice. Each driving period T of the protocol consists of four steps (each with duration of T/4), during which particle can only hop along the connected nearest-neighbour lattice sites. **b,** Illustration of chiral propagation along the lattice edge for a particle initially located at the left-bottom corner under the driving protocol in (a) after 8 driving periods (T1 to T8). (*11*)

The four-step driving protocol as descried in Fig.S1(a) can lead to anomalous edge states under proper conditions (*11*), which can be intuitively explained as following. As illustrated in Fig.S1(b), assuming a particle is initially located at the left-bottom corner of a finite lattice with edges and during each driving step, the hopping strength J and period T are coordinated such that the particle perfectly hops from its current site to its nearest-neighbour site. Under this condition, during the first step1, the particle will be transferred to its right black lattice site. In step2, as particle in the black site can only hop to the white site below it (see Fig.S1(a)), the particle will stay at the black site during step2 since the bottom edge effectively blocks this hopping path. In the next step3, the particle at the black site will be transferred to its right white site. In step4, as particle at white site can only hop to the black site below it and again the bottom edge blocks this hopping path, the particle will stay at the white site during step4. So, in one complete driving cycle T1, the particle effectively hops two lattice sites to its right. This process repeats until the particle hits the right-bottom corner, after which the particle will move up along the right vertical edge

under similar hopping constraints. The transport of the particle under 8 driving periods is illustrated in Fig.S1(b). So effectively an counterclockwise edge state can exist under the four-step driving protocol in Fig.S1(a). The above intuitive picture is based on the condition that the particle completely hops from its current site to its nearest-neighbour site during each step. However, this condition may not be satisfied such that there is a certain probability that the particle will stay at its original site after each step, which can lead to its transport to the bulk. In the following, the condition under which chiral edge states exist will be derived rigorously.

1) Floquet theory

For a time-dependent Hamiltonian $H(t)$ that is periodic in time with period T, i.e., $H(t) = H(t + T)$, the state of the system $|\psi(t)\rangle$ can be written as (*42*)

$$|\psi(t)\rangle = e^{-\frac{i\varepsilon t}{\hbar}} |\phi(t)\rangle \qquad (1.3)$$

with $|\phi(t)\rangle = |\phi(t + T)\rangle$ and ε is the quasienergy. The quasienergy spectrum is determined by the evolution operator in one period, i.e., $U(T) = \mathcal{T}\,[\exp(-\frac{i}{\hbar}\int_0^T H(t)dt)]$, where $\mathcal{T}$ denotes the time ordering because $H(t)$ and $H(t')$ in general do not commute. As $U(T)|\psi(0)\rangle = |\psi(T)\rangle = e^{-\frac{i\varepsilon T}{\hbar}} |\phi(T)\rangle = e^{-\frac{i\varepsilon T}{\hbar}}|\phi(0)\rangle = e^{-\frac{i\varepsilon T}{\hbar}} |\psi(0)\rangle$, we can calculate the quasienergy directly from the eigenvalues of $U(T)$. Because $e^{-i(\varepsilon+n\hbar\omega)T/\hbar} = e^{-i\varepsilon T/\hbar}$ for any n with $\omega = 2\pi/T$, we can restrict the quasienergy to the Brillouin zone $-\frac{\pi}{T} < \varepsilon \leq \frac{\pi}{T}$ similar to the crystal momentum of a system with lattice translational symmetry. For a system with both discrete time and spatial translational symmetries, the Floquet spectrum will form band $\varepsilon_n(k)$, where n is the band index and k the crystal momentum.

It is also convenient to define a static effective Hamiltonian H_{eff} through the Floquet operator $U(T)$ as H_{eff}: $U(T) = e^{-iH_{\text{eff}}T/\hbar}$. To get the effective Hamiltonian H_{eff}, we could define the branch cut of the logarithm to lie along the direction $e^{-i\epsilon T}$, i.e.,

$$\log_{\varepsilon} e^{i\phi} = i\phi, \quad -\varepsilon T - 2\pi < \phi \leq -\varepsilon T. \qquad (1.4)$$

which implies that

$$\log_{\varepsilon} e^{-i\varepsilon T + i0^-} = -i\varepsilon T, \quad \log_{\varepsilon} e^{-i\varepsilon T + i0^+} = -i\varepsilon T - 2\pi i \qquad (1.5)$$

Assuming that one can decompose the Floquet operator $U(T)$ as

$$U(T) = \sum_n \varepsilon_n(k) |\varepsilon_n(k)\rangle \langle \varepsilon_n(k)| \tag{1.6}$$

Then the effective Hamiltonian $H_{\text{eff}}^{\varepsilon}$ can be obtained as

$$H_{\text{eff}}^{\varepsilon} = \frac{i\hbar}{T} \sum_n \log_{\varepsilon}[\varepsilon_n(k)] |\varepsilon_n(k)\rangle \langle \varepsilon_n(k)| \tag{1.7}$$

From Eq. (1.3), we can get the evolution operator $U(T)$ as

$$U(T) = U_4\left(\frac{T}{4}\right) U_3\left(\frac{T}{4}\right) U_3\left(\frac{T}{4}\right) U_1\left(\frac{T}{4}\right) \tag{1.8}$$

where $H_1 = J\begin{pmatrix} 0 & e^{ik_x} \\ e^{-ik_x} & 0 \end{pmatrix}$, $H_2 = J\begin{pmatrix} 0 & e^{ik_y} \\ e^{-ik_y} & 0 \end{pmatrix}$, $H_3 = J\begin{pmatrix} 0 & e^{-ik_x} \\ e^{ik_x} & 0 \end{pmatrix}$, $H_4 = J\begin{pmatrix} 0 & e^{-ik_y} \\ e^{ik_y} & 0 \end{pmatrix}$, and $U_j\left(\frac{T}{4}\right) = e^{-iH_jT/4}$ $(j = 1,2,3,4)$.

Using $e^{i\alpha\begin{pmatrix} 0 & e^{i\theta} \\ e^{-i\theta} & 0 \end{pmatrix}} = \begin{pmatrix} \cos(\alpha) & \mathrm{i}e^{i\theta}\sin(\alpha) \\ \mathrm{i}e^{-i\theta}\sin(\alpha) & \cos(\alpha) \end{pmatrix}$, we have

$$U(T) = \begin{pmatrix} U_{11} & U_{12} \\ U_{21} & U_{22} \end{pmatrix} \tag{1.9}$$

in which

$$U_{11} = \frac{1}{8} e^{-2i(k_x+k_y)} \left(-e^{2ik_x} - e^{2ik_y} - 2e^{i(k_x+k_y)} + 6e^{2i(k_x+k_y)}\right.$$

$$\left. - e^{i(3k_x+k_y)} - e^{i(k_x+3k_y)} + \left(e^{ik_x} + e^{ik_y}\right)^2 \left(1 + e^{i(k_x+k_y)}\right) \cos(JT)\right)$$

$$U_{12} = ie^{-i(k_x+2k_y)}\left(e^{ik_x} + e^{ik_y} + e^{i(2k_x+k_y)} + e^{i(k_x+2k_y)}\right) \cos\left(\frac{JT}{4}\right) \sin\left(\frac{JT}{4}\right)$$

$$\left(-e^{ik_y} \cos\left(\frac{JT}{4}\right)^2 + e^{ik_x} \sin\left(\frac{JT}{4}\right)^2\right)$$

$$U_{21} = ie^{-i(2k_x+k_y)}\left(e^{ik_x} + e^{ik_y} + e^{i(2k_x+k_y)} + e^{i(k_x+2k_y)}\right) \cos\left(\frac{JT}{4}\right) \sin\left(\frac{JT}{4}\right)$$

$$\left(-e^{ik_x} \cos\left(\frac{JT}{4}\right)^2 + e^{ik_y} \sin\left(\frac{JT}{4}\right)^2\right)$$

$$U_{22} = \frac{1}{8} e^{-i(k_x+k_y)} \Big(-e^{2ik_x} - e^{2ik_y} - 2e^{2i(k_x+k_y)} + 6e^{i(k_x+k_y)} - e^{i(3k_x+k_y)} - e^{i(k_x+3k_y)} + \left(e^{ik_x} + e^{ik_y}\right)^2 \left(1 + e^{i(k_x+k_y)}\right) \cos(JT)\Big)$$

At $JT = 2n\pi$ $(n = 0,1,2, \dots)$, one can find $U(T) = \begin{pmatrix} 1 & 0 \\ 0 & 1 \end{pmatrix}$, which gives a flat band for the quasi-energy spectrum. The Floquet spectra of $U(T)$ when increasing JT are shown in Fig.S2, from which one can see that as JT increases, the width of the spectrum increases and as such, the spectrum gap at $\varepsilon = \pi$ decreases and closes at $JT = \pi$. When increasing JT further, the width of the spectrum shrinks and reduces to flat at $JT = 2\pi$, at which $U(T)$ is equal to the identity matrix.

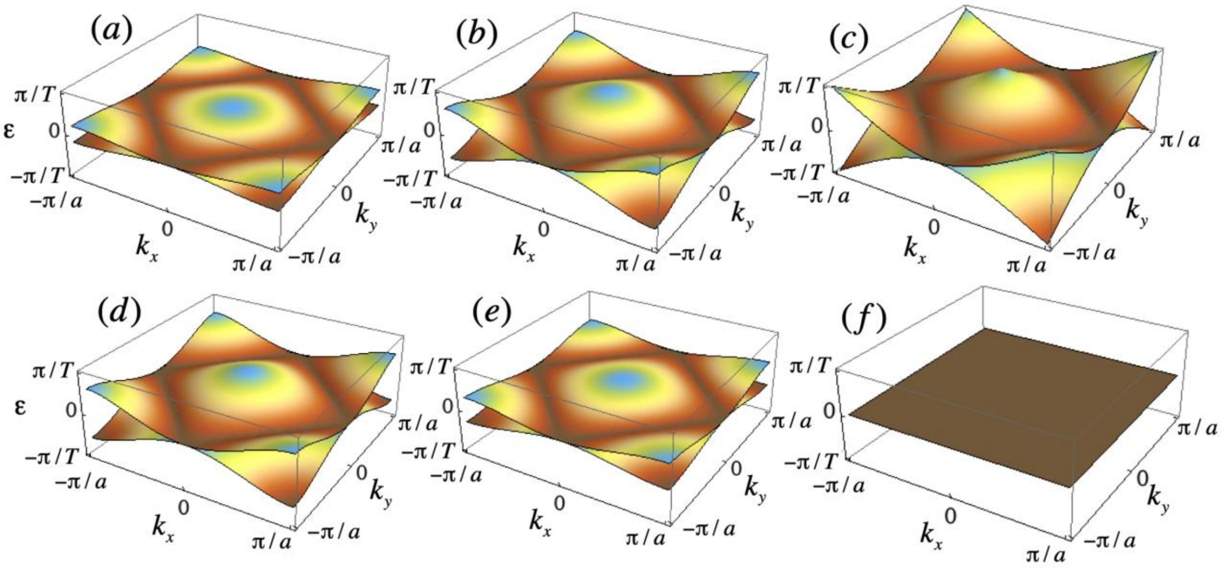

Fig. S2. Quasi-energy spectrum of the evolution operator $U(T)$ at different JT. **a,** $JT = 0.2\pi$; **b,** $JT = 0.6\pi$; **c,** $JT = 1.0\pi$; **d,** $JT = 1.4\pi$; **e,** $JT = 1.7\pi$; **f,** $JT = 2.0\pi$.

2) Topological invariant

Assuming that the Floquet spectrum has a gap over some fine interval $[\varepsilon - \Delta\varepsilon, \varepsilon + \Delta\varepsilon]$, for a model in a lattice with an open edge, the Floquet eigenstates with eigenvalues lying within this gap will correspond to edge states localized near the system edge. The number of these edge states could be determined by the bulk time evolution operator $U(k,t)$. As in general, $U(k,t) \neq U(k, t+T)$, one can not use $U(k,t)$ directly to construct the topological invariant. However, one can define the periodized time evolution operator (*43*)

$$V_\varepsilon(k,t) = U(k,t) e^{iH_{\text{eff}}^{\varepsilon}(k)t/\hbar} \qquad (1.10)$$

which satisfies $V_\varepsilon(k,t) = V_\varepsilon(k,t+T)$ as can be seen from

$$V_\varepsilon(k,t+T) = U(k,t+T)e^{\frac{iH_{\text{eff}}^{\varepsilon}(k)(t+T)}{\hbar}}$$

$$= U(k,t)\,U(k,T)\,e^{\frac{iH_{\text{eff}}^{\varepsilon}(k)T}{\hbar}}\,e^{\frac{iH_{\text{eff}}^{\varepsilon}(k)t}{\hbar}} = U(k,t)e^{\frac{iH_{\text{eff}}^{\varepsilon}(k)t}{\hbar}} = V_\varepsilon(k,t) \qquad (1.11)$$

As $V_\varepsilon(k,t)$ is periodic in (k_x, k_y, t), it defines a map from $S^1 \times S^1 \times S^1 \to U(2)$ and it is known that such map can be classified by a topological invariant (winding number),

$$W[V_\varepsilon(k,t)] = \frac{1}{8\pi^2}\int dt dk_x dk_y \text{Tr}(V^{-1}\partial_t V[V^{-1}\partial_{k_x}V, V^{-1}\partial_{k_y}V]) \qquad (1.12)$$

Note that the definition of the periodized time evolution operator is not unique. For example, a different definition as proposed in (*11*) is,

$$V_\varepsilon(k,t) = \begin{cases} U(k,2t), & \text{if } 0 \le t \le T/2 \\ e^{-iH_{\text{eff}}^{\varepsilon}(k)(2T-2t)/\hbar}, & \text{if } T/2 \le t \le T \end{cases} \qquad (1.13)$$

However, there two definitions are homotopic and as such their topological invariants defined above are equal. To see this, we can consider the following general map with parameter s,

$$G_\varepsilon(k,t,s) = \begin{cases} U(k,2t)e^{2iH_{\text{eff}}^{\varepsilon}(k)st/\hbar}, & \text{if } 0 \le t \le T/2 \\ e^{-2iH_{\text{eff}}^{\varepsilon}(k)(1-s)(T-t)/\hbar}, & \text{if } T/2 \le t \le T \end{cases} \qquad (1.14)$$

which satisfies $G_\varepsilon(k,0,s) = G_\varepsilon(k,T,s) = I$ and for $s = 0$, it reduces to the definition (1.13) and for $s = 1$, it reduces to

$$G_\varepsilon(k,t,1) = \begin{cases} U(k,2t)e^{2iH_{\text{eff}}^{\varepsilon}(k)t/\hbar}, & \text{if } 0 \le t \le T/2 \\ 1, & \text{if } T/2 \le t \le T \end{cases} \qquad (1.15)$$

which is homotopic with the definition (1.10). And thus the two definitions in (1.10) and (1.13) are homotopic.

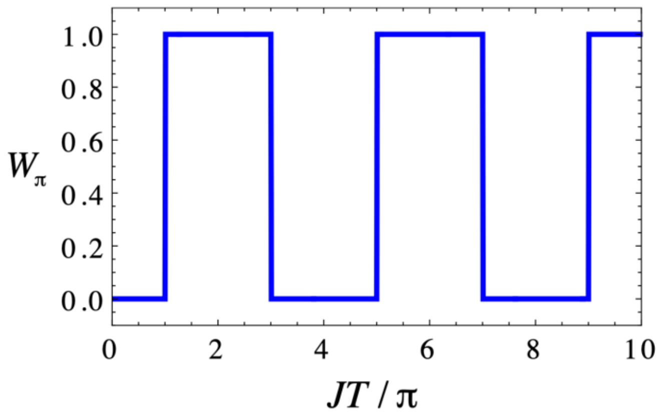

Fig. S3. Winding number at quasi-energy π as a function of JT.

The winding number of the system at $\varepsilon = \pi$ as a function of JT is shown in Fig.S3, which shows that the winding number is a periodic function of JT/π and has nontrivial value of 1 for the following JT,

$$W_\pi = 1, \quad 4n + 1 \leq \frac{JT}{\pi} \leq 4n + 3 \ (n = 0,1,2 \dots) \qquad (1.16)$$

The above result can be intuitively understood from the Rabi oscillation between two sites with the hopping strength J. Assuming the particle is at one site when the hopping J is turned on, the probability of the particle in this site as time evolves is $\cos{(Jt)^2}$. If at the end of one step time of $T/4$ the particle completely hops to its neighbouring site, then we can expect that the chiral edge states exist as explained in Fig.S1(b). This condition leads to $t = \frac{\pi}{2J}$, which is equal to $T/4$, i.e., $\frac{\pi}{2J} = \frac{T}{4} \to JT = 2\pi$. On the other hand, at the end of one step time of $T/4$, the particle remains at the original site, i.e.,$t = \frac{\pi}{J}$, then one can expect that there is no particle transport, i.e., the chiral edge state does not exist. In this case, $t = \frac{\pi}{J} = \frac{T}{4} \to JT = 4\pi$, which agrees with the winding number result shown in Fig. S3.

3) Floquet spectra for lattice with open edges

To show that the four-step driving protocol as defined in Fig. S1a can host the anomalous edge states within the quasienergy gap around π, we calculate the edge state spectra of the model in a geometry with open edges at different JT with the results shown in Fig. S4. When increasing JT from 0 to π, the bulk Floquet spectrum expands from $\varepsilon = 0$ to all the range of $\varepsilon \in [-\pi/T, +\pi/T]$

and no edge state is observed. However, when increasing JT further, one can see that edge states crossing $\pm\pi$ emerge and the bulk Floquet spectrum shrinks to the flat band at $\varepsilon = 0$ when $JT = 2\pi$. The results of the edge state spectra completely agree with the evolution of winding number shown in Fig. S3. The quasienergy gap at $\varepsilon = \pm\pi$ closes and reopens at $\frac{JT}{\pi} = 4n + 1$, and $\frac{JT}{\pi} = 4n + 3$ periodically, which is accompanied with topological phase transitions that lead to anomalous chiral edge states.

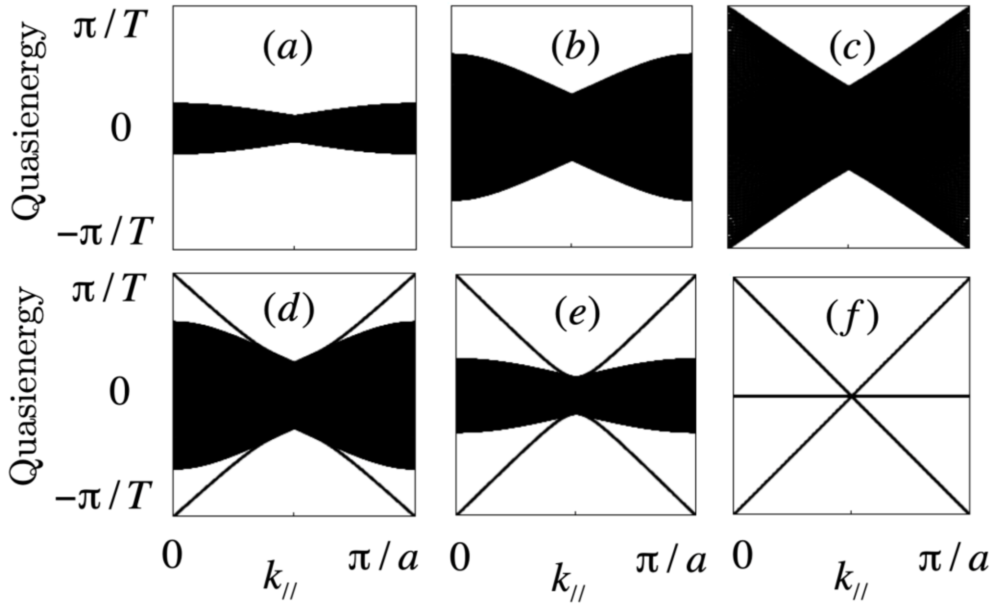

Fig. S4. Floquet spectra of a lattice in strip geometry with open edges at different JT. a, $JT = 0.2\pi$; **b,** $JT = 0.6\pi$; **c,** $JT = 1.0\pi$; **d,** $JT = 1.4\pi$; **e,** $JT = 1.7\pi$; **f,** $JT = 2.0\pi$.

Section 2: Mathematical principles of time-varying media modeling based on TDFIT

Notably, the time-varying media fall in the scope of dynamic electromagnetic (EM) problems, which, by introducing time as a new degree of freedom, enrich the design process and have garnered widespread attention. To date, researchers have made significant achievements in developing numerical methods for solving static EM problems. However, numerical simulation of dynamic EM problems remains a formidable challenge. Therefore, in this section, we introduce a computational EM method for solving time-varying media: the time-domain finite-integral theorem (TDFIT) *(45-52)*. By employing time-varying iterative coefficients to model time-varying media, we extend the powerful TDFIT method to solve dynamic EM problems.

The core of the algorithm is as follows:

$$\alpha_{ee}^{n} = \frac{[\bar{\varepsilon}(n \cdot \Delta t)/\Delta t - \bar{\sigma}(n \cdot \Delta t)/2]}{[\bar{\varepsilon}(n \cdot \Delta t)/\Delta t + \bar{\sigma}(n \cdot \Delta t)/2]} \tag{2.1}$$

$$\beta_{eh}^{n+1/2} = \frac{(l_k/\tilde{s}_k)}{[\bar{\varepsilon}(n \cdot \Delta t + \Delta t/2)/\Delta t + \bar{\sigma}(n \cdot \Delta t + \Delta t/2)/2]} \tag{2.2}$$

$$\alpha_{hh}^{n-1/2} = \frac{[\bar{\mu}(n \cdot \Delta t - \Delta t/2)/\Delta t - \bar{\kappa}(n \cdot \Delta t - \Delta t/2)/2]}{[\bar{\mu}(n \cdot \Delta t - \Delta t/2)/\Delta t + \bar{\kappa}(n \cdot \Delta t - \Delta t/2)/2]} \tag{2.3}$$

$$\beta_{he}^{n} = \frac{(\tilde{l}_k/s_k)}{[\bar{\mu}(n \cdot \Delta t)/\Delta t + \bar{\kappa}(n \cdot \Delta t)/2]} \tag{2.4}$$

Here, l_k and $\tilde{l}_k$ respectively denote the lengths of the edges of the primary and dual cells, whereas s_k and $\tilde{s}_k$ correspond to the areas of the primary and dual cells, respectively. Taking perfectly electrically conducting (PEC) materials as an example, the iterative coefficients are set as $\alpha_{ee} = 1$, $\beta_{eh} = 0$, $\alpha_{hh} = 1$, and $\beta_{he} = 0$. Herein, it is imperative to underscore that the introduction of time-varying iteration coefficients (namely, α_{ee}, β_{eh}, α_{hh}, and β_{he}) enables the numerical simulation of dynamic EM problems.

Section 3: The harmonic experimental results

In the harmonic experimental measurement, we use signal generator and spectrum analyser to test the harmonic electric-field response. More details about the experimental measurement are provided in Supplementary Section 5. Fig. S5a shows the experimental schematic diagram of the harmonic test, in which the position of the red five-pointed star is the excitation source and the position of the blue five-pointed star is the test probe point. Fig. S5b shows the results of the experimental test, where the x-axis is the modulation frequency and the y-axis is the test frequency. In the harmonic test, a single-frequency signal source is used for excitation (350MHz). When the modulation frequency is switched from 2-140MHz in 2MHz steps, the spectrum analyser measures the spectrum energy distribution of 150-500MHz. The test results show that the fundamental frequency signal has the strongest energy component, and the harmonic components gradually decrease from low order to high order. Experiments have verified that the time-varying topological metasurface (TVTM) has obvious harmonic characteristics.

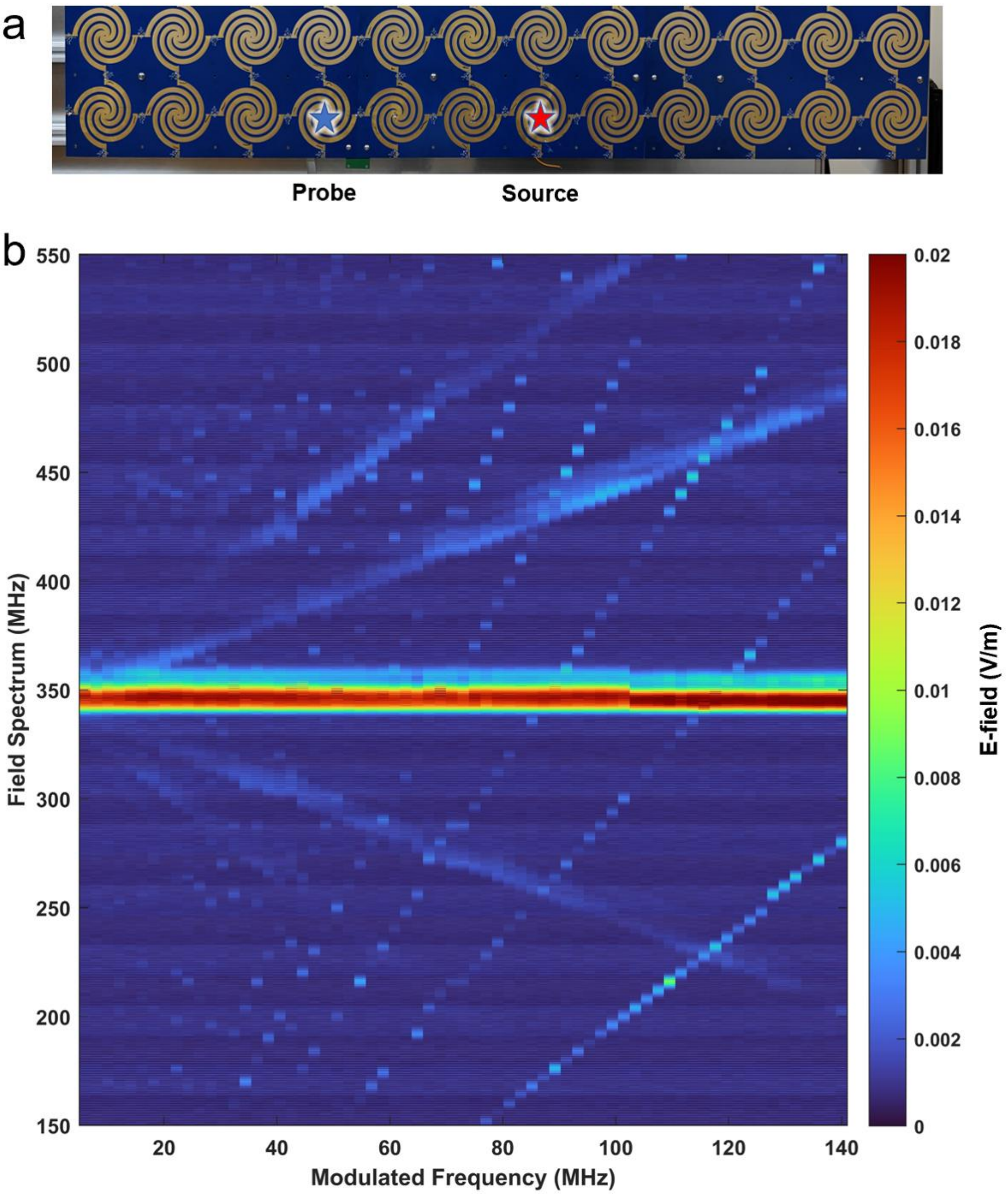

Fig. S5. The harmonic electric-field measured in experiments. a, The harmonic test illustration. The source is set at the central edge element (as red five-pointed star marked), while the test probe is set at the position three units away from the excitation source (as blue five-pointed star marked). **b,** The measured results as the modulated frequency is switched from 2MHz to 140 MHz (measurement step size of 2MHz).

Section 4: The detailed structure design of TVTM

Metasurface processing is based on mature printed circuit board technology. As shown in Fig. S6a, the metasurface is composed of 6 sub-boards and is fixed on the bracket through screws and aluminium profiles. Each SLSP arm contains two diodes, both of which can be independently programmed and controlled by the FPGA.

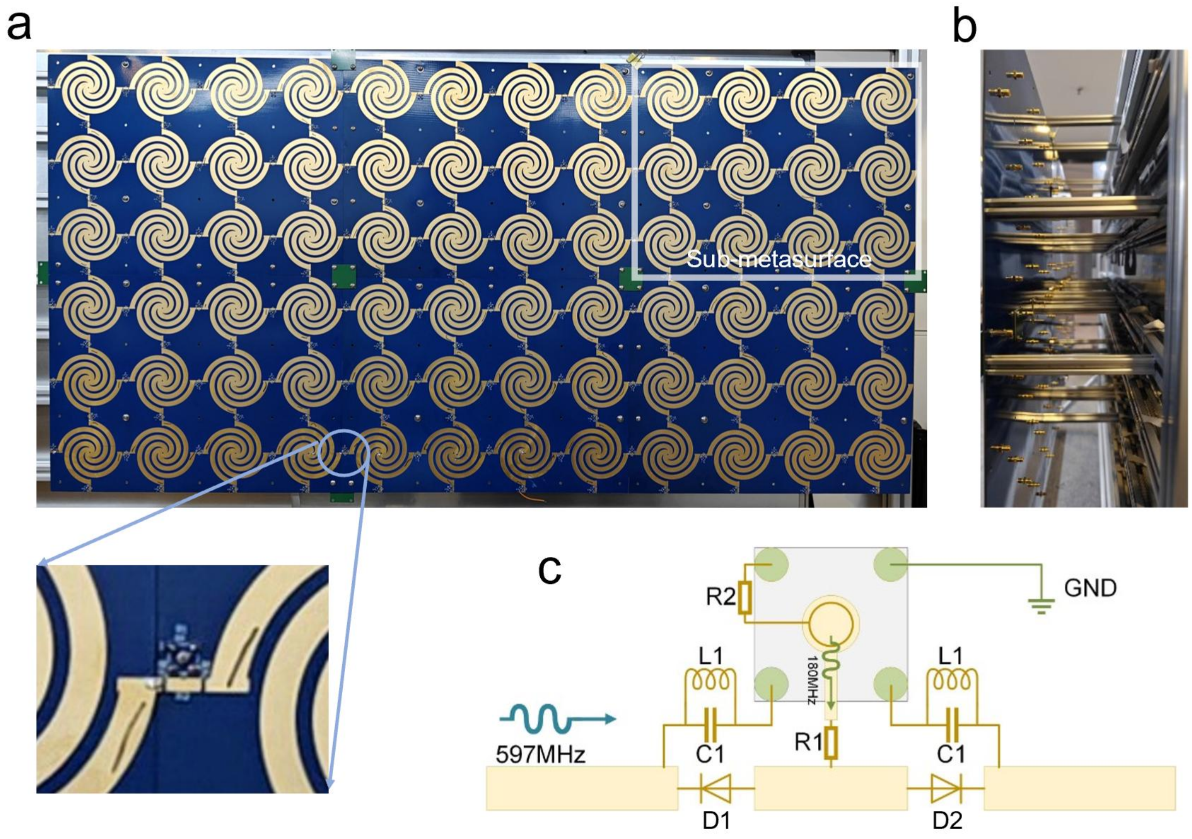

Fig. S6. The details of the metasurface fabrication design and configuration. a, Photo of the complete metasurface sample after installation. The 6 sub-metasurfaces are combined in the form of 2 rows and 3 columns, where the size of the sub-metasurfaces marked by white boxes. For interconnections between sub-metasurfaces, copper foils of adjacent metal sheets are soldered to achieve a conductive connection, as shown in the partially enlarged photo. **b,** Photos of metasurface metal fixed supports. The 6 sub-boards are flat and fixed together by aluminum alloy brackets. The coaxial connector on the back provides an impedance-matched transmission channel (coaxial cable) for high-speed control signals. **c,** Bias circuit design for high-speed switching channels. D1 and D2 are two symmetrically placed PIN diodes (MADP) with a common anode. The values of R1 and R2 are 510 ohms and 51 ohms respectively. L1 and C1 form a simple low-pass filter circuit. Considering that the significant frequency range of surface wave propagation is greater than 500MHz and the control signal frequency is less than 200MHz, the values of L1 and C1 are 20nH and 120pF.

In order to ensure the stability and flatness of the metasurface fixation, each sub-board (a total of 6 sub-boards) is fixed on a large aluminium alloy bracket through metal square pillars, as shown

in Fig. S6. The connections between daughter boards are further fixed with green plastic sheets to ensure flatness, as shown in Fig. S6a. Fig. S6b shows a photo of the metal posts connected and fixed. Fig. S6c shows the control circuit design of each arm, where the high-speed control signal (180MHz signal) is applied to the center of the arm, that is, the anode of the two PIN diodes, through a coaxial connector and a matching resistor (R1). The cathodes of the corresponding diodes on both sides of the arm are grounded through the LC bias circuit to isolate the excitation signal (the 597MHz signal).

Section 5: The measurement and control system of TVTM

The experimental testing in this work mainly involves two near-field experimental measurement methods: linear and harmonic near-field testing. As shown in Fig. S7a, first of all, a vector network analyzer is used for direct connection testing in the linear near-field test. The excitation frequency and the observation (test) frequency point are at the same frequency. It is mainly used to test the topological state field distribution; on the contrary, non- In the linear test, the excitation frequency and the observation (test) frequency point are different frequencies. Therefore, we use a signal source to output a single tone signal for excitation, and at the same time use a spectrum analyzer to observe the energy distribution at different frequency points. As the time modulation frequency of TVTM changes, the field distribution at fixed test points will show obvious harmonic characteristics. Fig. S7b shows a photo of the test environment, where the metasurface is mounted on a metal bracket. Each arm is connected to the FPGA control system through a coaxial cable. In addition, an oscilloscope monitors the output waveform frequency of the FPGA in real time.

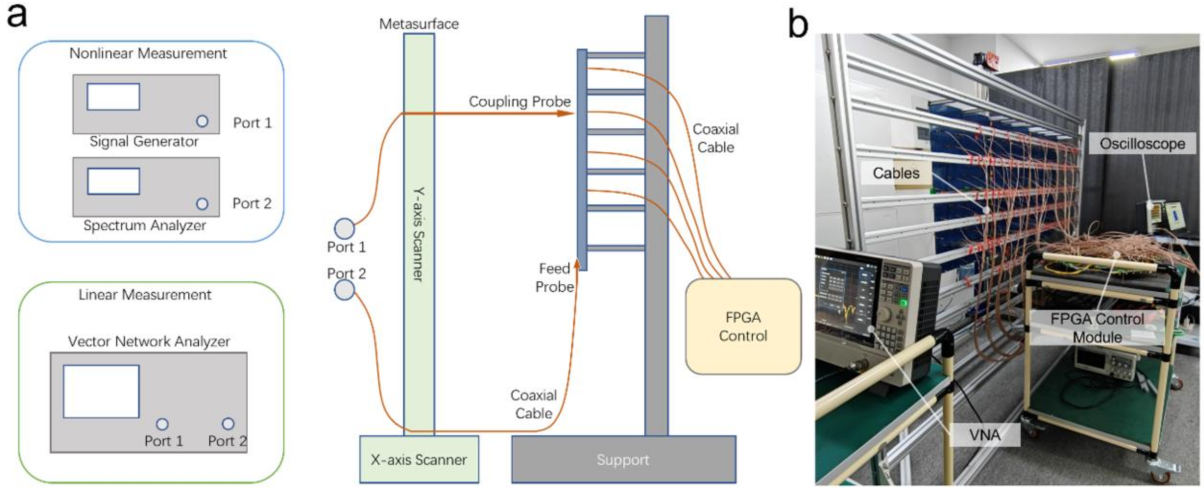

Fig. S7. The near-field measurement configuration. a, The near-field test platform consists of a set of two-dimensional platform scanning frames and supporting instruments. The metasurface is fixed on a support frame perpendicular to the ground, and the two-dimensional scanning frame scans the vertical plane. The two ports of the scanning system are respectively connected to the coupling probe (moving scanning) and the excitation probe (fixed position). The FPGA high-speed signal control module is installed on the back of the support frame and applies control signals through coaxial cables. Experimental measurements are mainly divided into linear and harmonic measurements. In the linear test, a vector network analyzer (VNA) is used to test the dual-port S parameters to obtain the energy distribution. In the harmonic test, a signal generator is used to excite the single tone signal, and the spectrum energy distribution is obtained through a spectrum analyzer. **b,** The near-field measurement and metasurface control system. The two-dimensional scanner is placed directly in front of the metasurface to test the surface electric field.

Section 6: The measured control signals of TVTM

In order to show the control signal waveforms in actual experimental tests, we show here 3 sets of measured waveform results, showing clockwise (CW) and counter-clockwise (CCW) switching at 150MHz, 180MHz and 200MHz respectively. As shown in Fig. S8, each color represents one of the timing cycles with a quarter duty cycle. We can observe that the high-frequency signal is not a perfect square wave waveform, mainly for the following reasons: 1) The operating frequency of the trigger of the driver board is limited, and the switching frequency in the experiment is close to the ultimate operating frequency of the D flip-flop. There is a certain delay in the turning on and off process, which affects the waveform; 2) There is a certain impedance mismatch in the control link. Impedance mismatch causes certain distortion during square wave signal propagation; 3) FPGA core frequency is limited. The FPGA core (AMD Zynq 7020) is 866MHz and cannot produce a perfect square wave signal frequency.

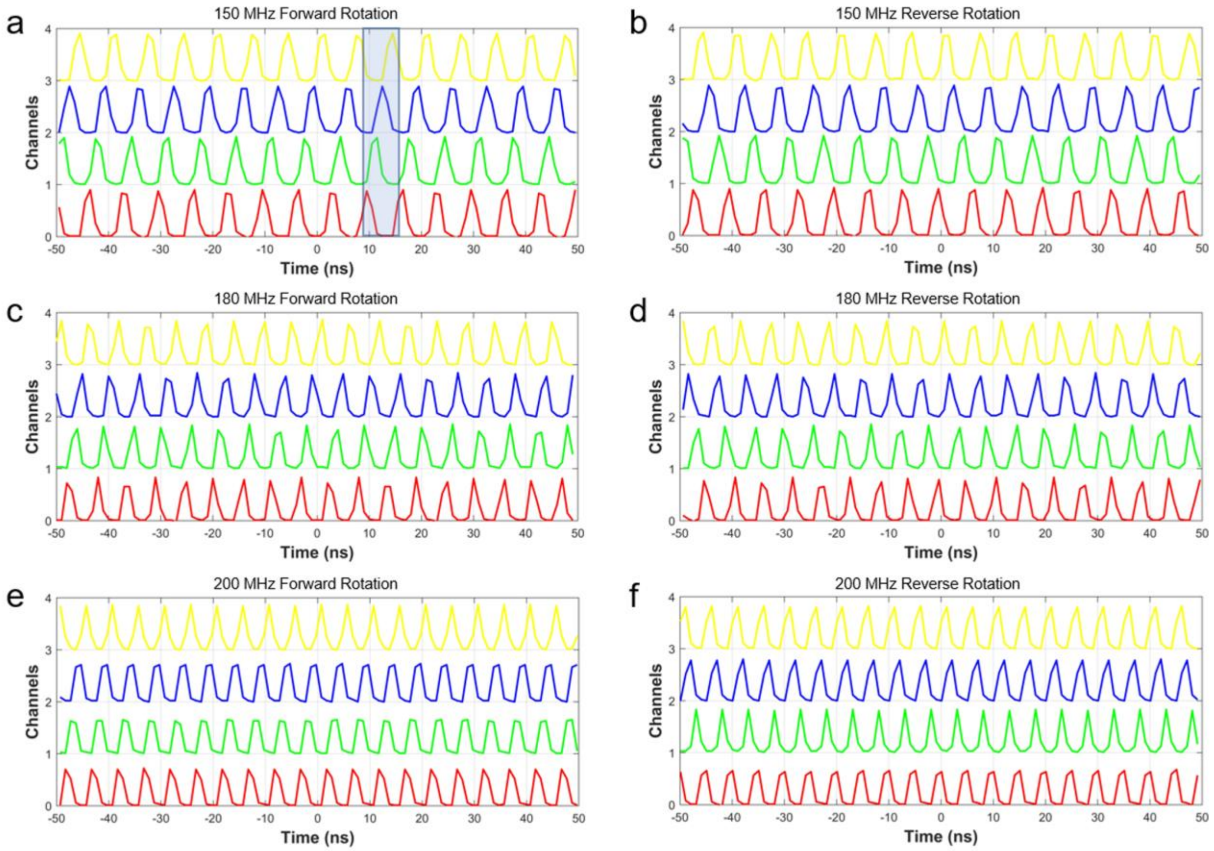

Fig. S8. The measured waveform of the high-speed signals. a-f, The high-speed control signal waveform measured by an oscilloscope (Siglent SDS2504X), in which the four channels respectively represent the switching control signals of the four connection channels of the control module (marked with yellow, green, red and blue respectively). The y-axis scale represents the signal voltage normalized intensity. In particular, **a and b,** The waveforms of 150 MHz CW and CCW switching, where one switching period of four channels is marked in a blue transparent square frame. **c and d,** The waveforms of 180 MHz CW and CCW switching. **e and f,** The waveforms of 200 MHz CW and CCW switching.

Section 7: Numerical calculation of light cones for the proposed metasurface

To explain the reason that the chiral edge states at the higher harmonics are not as obvious as those at the fundamental frequencies, we conducted numerical simulations of the light cone in this section. Figure S9a illustrates that the lattice period of our time-varying metasurface system is a = 60 mm, with the lattice geometry being square. Through rigorous numerical computations, we obtained the corresponding light cone presented in Fig. S9b, which further enhances the comprehension of the light cone characteristics within the 0-2 GHz range as depicted in Fig. 2d. The lower-order chiral edge state mode is entirely confined below the light cone, exhibiting

behavior typical of guided modes, where energy is more effectively localized at the surface, resulting in the highest energy intensity. As the frequency increases, the corresponding chiral edge states progressively emerge above the light cone, rendering them more prone to transitioning into radiative modes, thereby leading to a reduction in energy confinement.

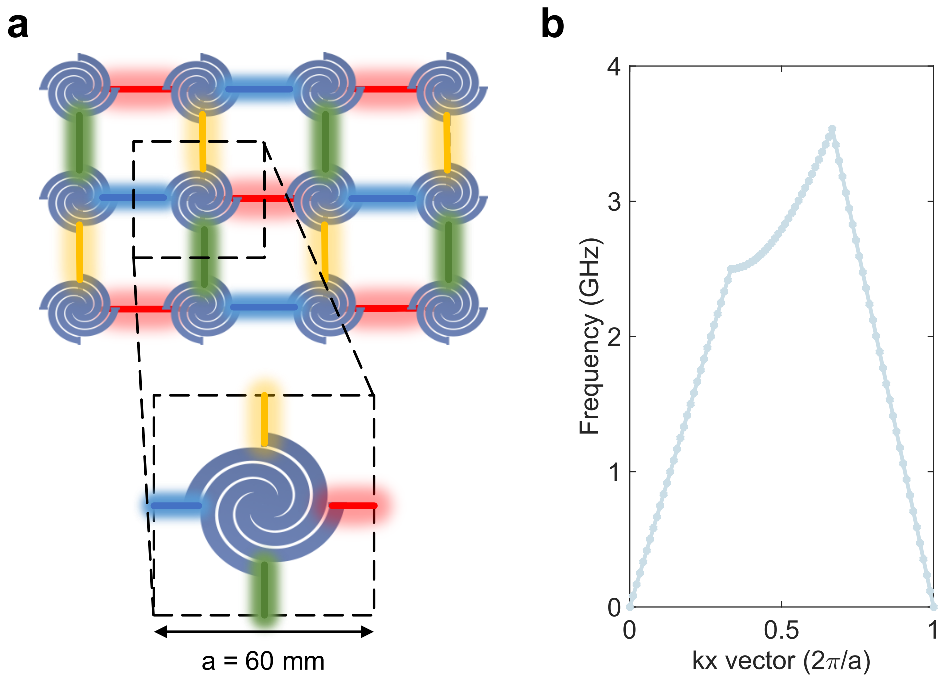

Fig. S9. Light cones of the proposed metasurface. a, Schematic of the time-varying metasurface lattice with period a = 60 mm. **b,** Numerical calculation of the light cone corresponding to the metasurface in **a.**

Section 8: Numerical modeling of the equivalent time-varying medium

In this section, we present an innovative method for numerically simulating a realistic PIN diode by modeling it as an equivalent time-varying medium (*51*). Currently, PIN diode simulations predominantly utilize RLC-based equivalent circuits or large-signal equivalent circuit models. A significant limitation of these conventional techniques is their inability to accurately simulate the dynamic switching behavior and transient responses of PIN diodes, as they are primarily restricted to modeling either the conductive or non-conductive states.

To address this limitation, we propose a novel equivalent time-varying medium model, as depicted in Fig. S10a. The essence of this model resides in the introduction of the conductivity as an electromagnetic parameter to simulate the behavior of the PIN diode. Specifically, the conductivity is assigned a value of 5.8×10^7 S/m in the conductive state and 0 S/m in the non-

conductive state. As depicted in Fig. S10b, the electromagnetic field distributions in a supercell are presented, where four PIN diodes are sequentially activated at equidistant time intervals. The results reveal that a current density distribution is only observable when the PIN diodes are in their conductive state, namely switch-on state, thereby clearly indicating the connectivity of the diodes. This observation not only substantiates the effectiveness of the proposed modeling approach but also lays an empirical foundation for further exploration of the electromagnetic behavior of time-varying media.

Furthermore, it highlights the pivotal role of incorporating time-varying conductivity in influencing system behavior. The temporal variation of conductivity inevitably induces dynamic alterations in the dielectric constant, which are particularly critical in our TDFIT numerical method as they directly impact the accuracy and stability of solving the entire time-varying system. Additionally, leveraging this modeling framework, we can accurately analyze the momentum-space energy band structure diagrams of the proposed topological metasurfaces, as illustrated in Fig. S11.

In summary, our time-varying medium modeling approach introduces a novel and systematic methodology for analyzing and comprehending time-varying systems, thereby substantially advancing related research. This framework not only extends the boundaries of conventional circuit models but also establishes a robust foundation for future research initiatives, particularly in the domains of rapid response and dynamic electromagnetic characteristic analysis. We expect that this method will reveal its full potential across a wider range of applications and make significant contributions to the progress of electromagnetic research and technology.

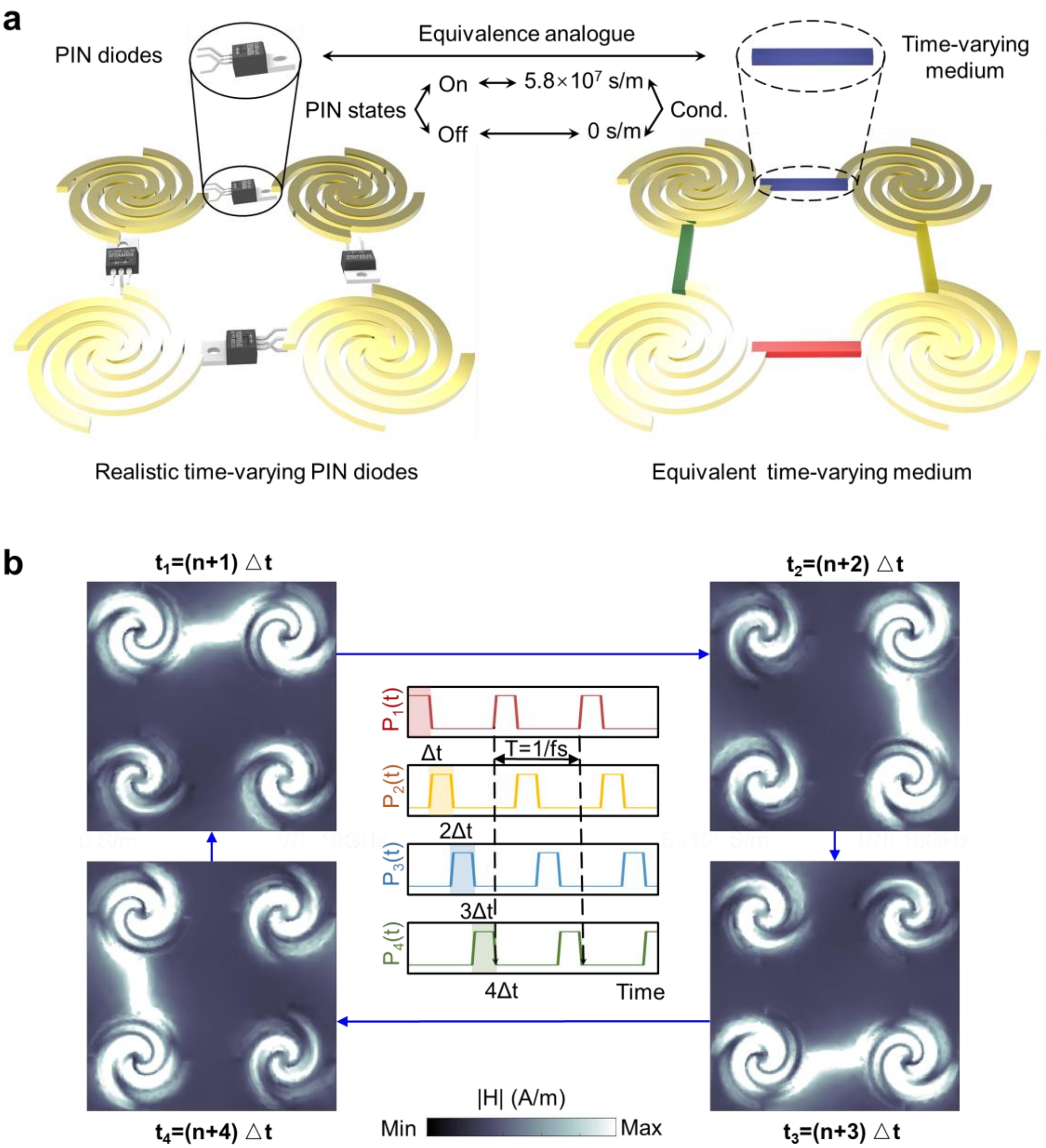

Fig. S10. Numerical modeling of the equivalent time-varying medium. a, Schematic diagram of the realistic PIN diode and its equivalent time-varying medium model. **b**, Electromagnetic field distributions in a in a supercell, where four PIN diodes are sequentially activated at equidistant time intervals. The results reveal that a current density distribution is only observable when the PIN diodes are in their conductive state, namely switch-on state, thereby clearly indicating the connectivity of the diodes.

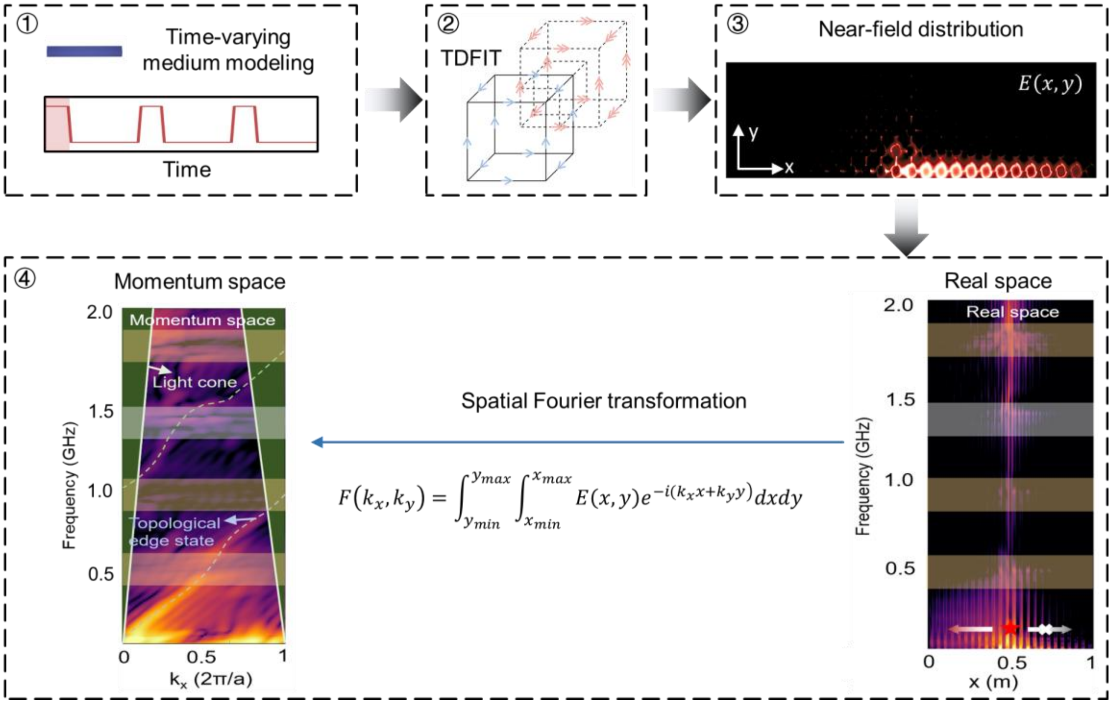

Fig. S11. Flowchart for simulating a realistic time-varying metasurface to obtain the band structures.

Section 9: Reprogrammable time-varying topological states

While the time-modulation protocol studied above is the same for all unit cells, the proposed time-varying metasurface in principle can support any time modulation patterns, which allows to realize new functions for diverse applications. To demonstrate this feature, we further propose a programmable time-varying topological metasurface with inhomogeneous modulation patterns, as shown in Fig. S12a, where each unit cell is independently switched to the digital states ‘0’ and ‘1’. The binary code ‘0’ represents the state when all PIN diodes in the unit cell are always closed, maintaining a static state; while the binary code ‘1’ refers to the state when all PIN diodes (red, yellow, blue, and green) in the unit cell are switched on and off sequentially following either a CW or CCW direction. Applying different encoding sequences to each unit cell by FPGA in real time, we can dynamically reconfigure the unit cells encoded by ‘0’ and ‘1’ into arbitrary patterns, which enable the on-demand manipulations of electromagnetic wave propagation along any prescribed trajectory. Physically, when the four PIN diodes in the programmable unit cell are turned off and maintained in the cut-off state, the unit cell is ‘invisible’ to electromagnetic waves at that moment, resembling a shielded state. This could be understood from the quasi-energy spectrum in the momentum space at low modulation frequency (see e.g., Fig. 2b), where only trivial bulk states

exist. On the other hand, when the programmable unit cells are switched at high frequency of 450MHz, the topological edge states emerge during this time (see Fig. 2d), which can be used to encode the binary code for the ‘1’ unit cell. As a result, by controlling the states of the PIN diodes, the binary expression of the topological phase transition between the two binary encoded programmable unit cells is effectively converted into a digital expression, enabling the realization of a digitized and dynamically controllable topological propagation interface.

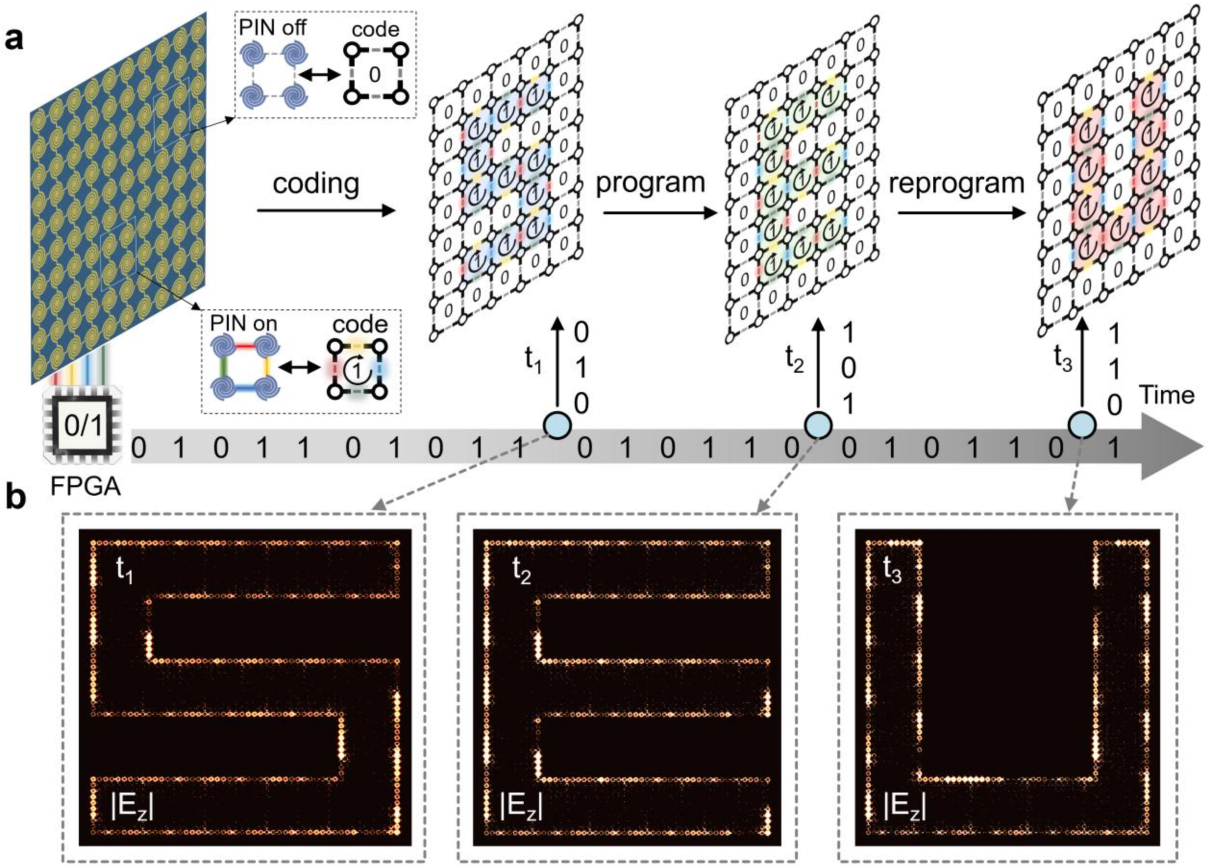

Fig. S12. Programmable time-varying topological metasurface with the reconfigurable propagation routes. a, Schematic illustration of the ultra-fast programmable time-varying topological metasurface, where each unit cell has two digital states of ‘0’ and ‘1’. The ‘0’ state (static state) is realized when all connecting PIN diodes are switched off, while the ‘1’ state (dynamic state) is realized via the four-step sequential driving protocol, which are electrically controlled by FPGA. To demonstrate the reconfigurability of the programmable metasurface for realizing arbitrary topological propagation routes, three encoding patterns are taken as examples for the propagation routes by emulating three different ASCII sequences (‘S’, ‘E’, ‘U’). **b,** Simulated near-field distributions of the programmable time-varying metasurface (54×54 unit cells) emulating the three letters (‘S’, ‘E’, ‘U’).

To explicitly show the feasibility to reconfigure the propagation paths following the above idea, we encode three ASCII sequences (‘S’, ‘E’, ‘U’) accordingly, as schematically shown in Figs.

S12a. Note that different topological propagation paths are encoded to dynamically discretize the continuous-wave analog input signals into different digital signals at the output port. While the schematics in Fig. S12a only have 5×7 unit cells for illustration purpose, the real metasurface emulating the three letters ('S', 'E', 'U') has a size of 54×54 units in numerical calculations. The simulated near-field distributions of $|E_z|$ are given in Fig. S12b, where three letters are clearly observed. The results also show that the electromagnetic waves propagate only in a unidirectional manner along the edge of the path. In addition to the feature of unidirectional propagation, immunity of the propagation against sharp bending corners is also observed. We confirm that the propagation is robust in the presence of significant defects and high disorders. Due to its flexible programmability, many existing photonic topological functions can be easily implemented on the proposed programable time-varying platform.

Section 10: The mathematical framework of harmonic Floquet chiral edge states

Time-varying metasurfaces represent a subcategory of four-dimensional metamaterials. In conventional nonlinear optics research, frequency conversion relies predominantly on nonlinear media. However, with the introduction of the temporal dimension, frequency manipulation is no longer confined to nonlinear materials and can also be achieved through temporal modulation.

We will detail the mechanisms of our time-varying metasurfaces in this section. To better illustrate the spectral components generated by the introduction of pump frequencies, we begin with Maxwell's equations, as represented by (*53*):

$$\begin{cases} \nabla \cdot \boldsymbol{D} = \rho \\ \nabla \cdot \boldsymbol{B} = 0 \\ \nabla \times \boldsymbol{H} = \boldsymbol{J} + \dfrac{\partial \boldsymbol{D}}{\partial t} \\ \nabla \times \boldsymbol{E} = -\dfrac{\partial \boldsymbol{B}}{\partial t} \end{cases} \tag{10.1}$$

In our time-varying system, since surface waves are being transmitted, we can derive the expression for the electric field of surface waves based on the aforementioned equations (*52*). We assume that the surface wave propagates along the x-axis, with the interface located at $z = 0$, where the metal occupies the region $z < 0$ and the dielectric material occupies $z > 0$.

$$\begin{cases} E_m(x, z, t) = E_0 e^{i(kx-\omega t)} e^{k_m z} \ (z < 0) \\ E_d(x, z, t) = E_0 e^{i(kx-\omega t)} e^{-k_d z} \ (z > 0) \end{cases} \tag{10.2}$$

Here, E_m indicates the electric field on the metal side, while E_d represents the electric field on the dielectric side. E_0 is the amplitude vector of the electric field, k is the propagation constant of the surface wave, ω is the angular frequency, and k_m and k_d are the vertical attenuation constants in the metal and dielectric, respectively. In the metal, the presence of free electrons causes the electric field to induce a current density. Furthermore, considering the time-varying nature of the system, the surface current density $\boldsymbol{J}_s$ can be expressed as (*52*):

$$\boldsymbol{J}_s(t) = \sigma(t)\boldsymbol{E}(t) \tag{10.3}$$

From this equation, we see that there exists a crucial time-varying parameter, the conductivity, which can be expressed within a single Floquet driving cycle as:

$$\sigma(1,2,3,4) = \begin{cases} (1,0,0,0), 0 \le t \le \frac{T}{4} \\ (0,1,0,0), \frac{T}{4} < t \le \frac{T}{2} \\ (0,0,1,0), \frac{T}{2} < t \le \frac{3T}{4} \\ (0,0,0,1), \frac{3T}{4} < t \le T \end{cases} \tag{10.4}$$

It is important to note that in this expression, we have normalized the conductivity of the system, and $\sigma(1,2,3,4)$ denotes the conductivity of the four different connecting arms of a square lattice. By analyzing the surface current density in this context, we find that the current density undergoes periodic changes in a clockwise or counterclockwise manner during the four equal driving time steps within a single Floquet driving cycle, primarily reflected in its phase characteristics. Considering any two adjacent time steps within the same Floquet driving cycle, the current density has a phase shift of $\frac{\pi}{2}$, which manifests as an orthogonal transformation of polarization under the same excitation. Similarly, the current densities that are two time-steps apart exhibit a phase shift of π without a change in polarization. We can provide the following expression:

$$\boldsymbol{J}_s(t) = \begin{cases} \boldsymbol{J}_0 e^{-i\omega t} e^{i0}, 0 \leq t \leq \frac{T}{4} \\ \boldsymbol{J}_0 e^{-i\omega t} e^{i\frac{\pi}{2}}, \frac{T}{4} < t \leq \frac{T}{2} \\ \boldsymbol{J}_0 e^{-i\omega t} e^{i\pi}, \frac{T}{2} < t \leq \frac{3T}{4} \\ \boldsymbol{J}_0 e^{-i\omega t} e^{i\frac{3\pi}{2}}, \frac{3T}{4} < t \leq T \end{cases} \quad (10.5)$$

where $\boldsymbol{J}_0$ represents the surface current under a static modulation condition. Based on equations (10.3) and (10.5), we can further derive the expression for the surface electric field:

$$\boldsymbol{E}_s(t) = \begin{cases} \boldsymbol{E}_0 e^{-i\omega t} e^{i0}, 0 \leq t \leq \frac{T}{4} \\ \boldsymbol{E}_0 e^{-i\omega t} e^{i\frac{\pi}{2}}, \frac{T}{4} < t \leq \frac{T}{2} \\ \boldsymbol{E}_0 e^{-i\omega t} e^{i\pi}, \frac{T}{2} < t \leq \frac{3T}{4} \\ \boldsymbol{E}_0 e^{-i\omega t} e^{i\frac{3\pi}{2}}, \frac{3T}{4} < t \leq T \end{cases} \quad (10.6)$$

Next, we illustrate the nonlinear modulation process under monochromatic incidence using the proposed time-varying topological metasurface. The modulation rate $\eta(t)$ of the metasurface is defined as a periodic signal, with the incident wave denoted as $E_i(t)$. Thus, the surface wave $E_s(t)$ can be expressed as the product of $\eta(t)$ and $E_i(t)$:

$$E_s(t) = E_i(t) \cdot \eta(t) \quad (10.7)$$

Consequently, the Fourier transform can be written as:

$$E_s(f) = E_i(f) * \eta(f) \quad (10.8)$$

The modulation rate function over a single period is defined as:

$$\eta(t) = \begin{cases} e^{-i\omega t} e^{i0}, 0 < t \leq \frac{T}{4} \\ e^{-i\omega t} e^{i\frac{\pi}{2}}, \frac{T}{4} < t \leq \frac{T}{2} \\ e^{-i\omega t} e^{i\pi}, \frac{T}{2} < t \leq \frac{3T}{4} \\ e^{-i\omega t} e^{i\frac{3\pi}{2}}, \frac{3T}{4} < t \leq T \end{cases} \quad (10.9)$$

The periodic modulation rate function $\eta(t)$ can also be expressed in the form of a Fourier series as follows:

$$\eta(t) = \sum_{n=-\infty}^{\infty} c_n \, e^{in2\pi t/T} \tag{10.10}$$

where

$$c_n = \frac{1}{T}\int_{-\frac{T}{2}}^{\frac{T}{2}} \eta(t) e^{-in2\pi t/T} dt$$

$$= \frac{1}{T}\left(\begin{array}{c} \int_{-\frac{T}{2}}^{-\frac{T}{4}} e^{i\pi} e^{-i\omega t} e^{-in2\pi t/T} dt + \int_{-\frac{T}{4}}^{0} e^{i\frac{3\pi}{2}} e^{-i\omega t} e^{-in2\pi t/T} dt + \\ \int_{0}^{\frac{T}{4}} e^{i0} e^{-i\omega t} e^{-in2\pi t/T} dt + \int_{\frac{T}{4}}^{\frac{T}{2}} e^{i\frac{\pi}{2}} e^{-i\omega t} e^{-in2\pi t/T} dt \end{array} \right)$$

$$= \frac{1}{T}\left(\begin{array}{c} -\left.\frac{e^{-i\omega t} e^{-in2\pi t/T}}{-i(n\,2\pi/T + \omega)}\right|_{-\frac{T}{2}}^{-\frac{T}{4}} - i\left.\frac{e^{-i\omega t} e^{-in2\pi t/T}}{-i(n\,2\pi/T + \omega)}\right|_{-\frac{T}{4}}^{0} + \\ \left.\frac{e^{-i\omega t} e^{-in2\pi t/T}}{-i(n\,2\pi/T + \omega)}\right|_{0}^{\frac{T}{4}} + i\left.\frac{e^{-i\omega t} e^{-in2\pi t/T}}{-i(n\,2\pi/T + \omega)}\right|_{\frac{T}{4}}^{\frac{T}{2}} \end{array} \right)$$

$$= \frac{1}{T}\left(\begin{array}{c} \frac{e^{in\pi} e^{i\omega T/2} - 1 - 2i\sin\left(\frac{n\pi}{2} + \frac{\omega T}{4}\right)}{-i(n\,2\pi/T + \omega)} + \\ \frac{e^{-in\pi} e^{-i\omega T/2} - 1 + 2i\sin\left(\frac{n\pi}{2} + \frac{\omega T}{4}\right)}{-(n\,2\pi/T + \omega)} \end{array} \right)$$

$$= \frac{(i+1)\left(2\sin\left(\frac{n\pi}{2} + \frac{\omega T}{4}\right) + 1 - \sin\left(n\pi + \frac{\omega T}{2}\right) - \cos\left(n\pi + \frac{\omega T}{2}\right)\right)}{i(n\,2\pi/T + \omega)} \; (n \neq 0) \tag{10.11}$$

$$c_0 = \frac{1}{T}\int_{-\frac{T}{2}}^{\frac{T}{2}} \eta(t) dt = \frac{1}{T}\left(\begin{array}{c} \int_{-\frac{T}{2}}^{-\frac{T}{4}} e^{i\pi} e^{-i\omega t} dt + \int_{-\frac{T}{4}}^{0} e^{i\frac{3\pi}{2}} e^{-i\omega t} dt + \\ \int_{0}^{\frac{T}{4}} e^{i0} e^{-i\omega t} dt + \int_{\frac{T}{4}}^{\frac{T}{2}} e^{i\frac{\pi}{2}} e^{-i\omega t} dt \end{array} \right) \neq 0 \tag{10.12}$$

We derive the following expression:

$$c_n = \frac{(i+1)\left(2\sin\left(\frac{n\pi}{2} + \frac{\omega T}{4}\right) + 1 - \sin\left(n\pi + \frac{\omega T}{2}\right) - \cos\left(n\pi + \frac{\omega T}{2}\right)\right)}{i(n\,2\pi/T + \omega)} \tag{10.13}$$

For spatially invariant systems, the periodic modulation rate function $\eta(t)$ can be represented as a sum of Fourier series, as shown in Equation (10.10). Its Fourier transform can be expressed as:

$$\eta(f) = 2\pi \sum_{n=-\infty}^{\infty} c_n\, \delta(f - nf_0)$$

$$= 2\pi \sum_{n=-\infty}^{\infty} \frac{(i+1)\left(2\sin\left(\frac{n\pi}{2} + \frac{\omega T}{4}\right) + 1 - \sin\left(n\pi + \frac{\omega T}{2}\right) - \cos\left(n\pi + \frac{\omega T}{2}\right)\right)}{i(n\,2\pi/T + \omega)}$$
$$\delta(f - nf_0) \tag{10.14}$$

where $f_0 = \frac{1}{T}$ is the modulation frequency, and c_n denotes the Fourier series coefficients at nf_0. The temporal modulation rate induces a frequency conversion effect on the incident wave, introducing a series of higher-order harmonics at nf_0. Under the excitation of a monochromatic signal $E_i(f)$ at frequency f_c, the output signal can be represented as:

$$E_s(f) = \sum_{n=-\infty}^{\infty} \frac{(i+1)\left(2\sin\left(\frac{n\pi}{2} + \frac{\pi f_c T}{2}\right) + 1 - \sin(n\pi + \pi f_c T) - \cos(n\pi + \pi f_c T)\right)}{i(nf_0 + f_c)}$$
$$E_i(f - f_c - nf_0) \tag{10.15}$$

This can be further rewritten as:

$$E_s(f) = c_0 E_i(f - f_c) + \sum_{k=1}^{\infty} [c_n E_i(f - f_c - nf_0) + c_{-n} E_i(f - f_c + nf_0)] \tag{10.16}$$

Finally, from the above equations, we can obtain the fundamental and harmonic intensities:

$$|c_n| = \sqrt{2} \left| \frac{\left(2\sin\left(\frac{n\pi}{2} + \frac{\pi f_c T}{2}\right) + 1 - \sin(n\pi + \pi f_c T) - \cos(n\pi + \pi f_c T)\right)}{(nf_0 + f_c)} \right| \tag{10.17}$$

If another driving protocol with opposite rotational direction is employed, the corresponding results can be similarly derived. In this case, Equation (10.9) can be rewritten as:

$$\eta(t) = \begin{cases} e^{-i\omega t}e^{i\frac{3\pi}{2}}, 0 < t \leq \frac{T}{4} \\ e^{-i\omega t}e^{i\pi}, \frac{T}{4} < t \leq \frac{T}{2} \\ e^{-i\omega t}e^{i\frac{\pi}{2}}, \frac{T}{2} < t \leq \frac{3T}{4} \\ e^{-i\omega t}e^{i0}, \frac{3T}{4} < t \leq T \end{cases} \tag{10.18}$$

Thus, the expression for c_n can be given as:

$$c_n = \frac{1}{T}\int_{-\frac{T}{2}}^{\frac{T}{2}} \eta(t) e^{-in2\pi t/T} dt$$

$$= \frac{1}{T}\left(\begin{array}{c} \int_{-\frac{T}{2}}^{-\frac{T}{4}} e^{i\frac{\pi}{2}} e^{-i\omega t} e^{-in2\pi t/T} dt + \int_{-\frac{T}{4}}^{0} e^{i0} e^{-i\omega t} e^{-in2\pi t/T} dt + \\ \int_{0}^{\frac{T}{4}} e^{i\frac{3\pi}{2}} e^{-i\omega t} e^{-in2\pi t/T} dt + \int_{\frac{T}{4}}^{\frac{T}{2}} e^{i\pi} e^{-i\omega t} e^{-in2\pi t/T} dt \end{array} \right)$$

$$= \frac{1}{T}\left(\begin{array}{c} i \frac{e^{-i\omega t} e^{-in2\pi t/T}}{-i(n\,2\pi/T + \omega)}\bigg|_{-\frac{T}{2}}^{-\frac{T}{4}} + \frac{e^{-i\omega t} e^{-in2\pi t/T}}{-i(n\,2\pi/T + \omega)}\bigg|_{-\frac{T}{4}}^{0} - \\ i \frac{e^{-i\omega t} e^{-in2\pi t/T}}{-i(n\,2\pi/T + \omega)}\bigg|_{0}^{\frac{T}{4}} - \frac{e^{-i\omega t} e^{-in2\pi t/T}}{-i(n\,2\pi/T + \omega)}\bigg|_{\frac{T}{4}}^{\frac{T}{2}} \end{array} \right)$$

$$= \frac{1}{T}\left(\begin{array}{c} -i \frac{e^{in\pi} e^{i\omega T/2} - 1 - 2i \sin\left(\frac{n\pi}{2} + \frac{\omega T}{4}\right)}{-i(n\,2\pi/T + \omega)} + \\ i \frac{e^{-in\pi} e^{-i\omega T/2} - 1 + 2i \sin\left(\frac{n\pi}{2} + \frac{\omega T}{4}\right)}{-(n\,2\pi/T + \omega)} \end{array} \right)$$

$$= \frac{(i+1)\left(2\sin\left(\frac{n\pi}{2} + \frac{\omega T}{4}\right) - 1 - \sin\left(n\pi + \frac{\omega T}{2}\right) + \cos\left(n\pi + \frac{\omega T}{2}\right)\right)}{i(n\,2\pi/T + \omega)} \; (n \neq 0) \tag{10.19}$$

We can arrive at the final result:

$$c_n = \frac{(i+1)\left(2\sin\left(\frac{n\pi}{2} + \frac{\omega T}{4}\right) - 1 - \sin\left(n\pi + \frac{\omega T}{2}\right) + \cos\left(n\pi + \frac{\omega T}{2}\right)\right)}{i(n\,2\pi/T + \omega)} \tag{10.20}$$

In this scenario, we update Equation (10.15) to:

$$E_s(f) = \sum_{n=-\infty}^{\infty} \frac{(i+1)\left(2\sin\left(\frac{n\pi}{2}+\frac{\pi f_c T}{2}\right) - 1 - \sin(n\pi + \pi f_c T) + \cos(n\pi + \pi f_c T)\right)}{i(nf_0 + f_c)} E_i(f - f_c - nf_0) \quad (10.21)$$

Therefore, we can determine the fundamental and harmonic intensities as:

$$|c_n| = \sqrt{2}\left|\frac{\left(2\sin\left(\frac{n\pi}{2}+\frac{\pi f_c T}{2}\right) - 1 - \sin(n\pi + \pi f_c T) + \cos(n\pi + \pi f_c T)\right)}{(nf_0 + f_c)}\right| \quad (10.22)$$

Section 11: The principle of edge state generation

In this section, we introduce the fundamental physical mechanisms governing the emergence of edge states and the pivotal factors that modulate their characteristics, as illustrated in Fig. S13. Initially, in the absence of time-varying modulation, only one intrinsic bulk-state region (depicted by black region) is present, and there are not any topological edge states. When employing traditional time-varying scheme (such as periodic square wave switching), all PIN diodes maintain synchronized switching states. In this case, several harmonic bulk-state regions are generated, leading to a frequency conversion phenomenon. However, the topological edge states cannot be generated in this traditional time-varying scheme. Therefore, although the harmonic bulk states can be generated in any time-varying system, it cannot be guaranteed that topological edge states can be generated in any wave system that is time modulated.

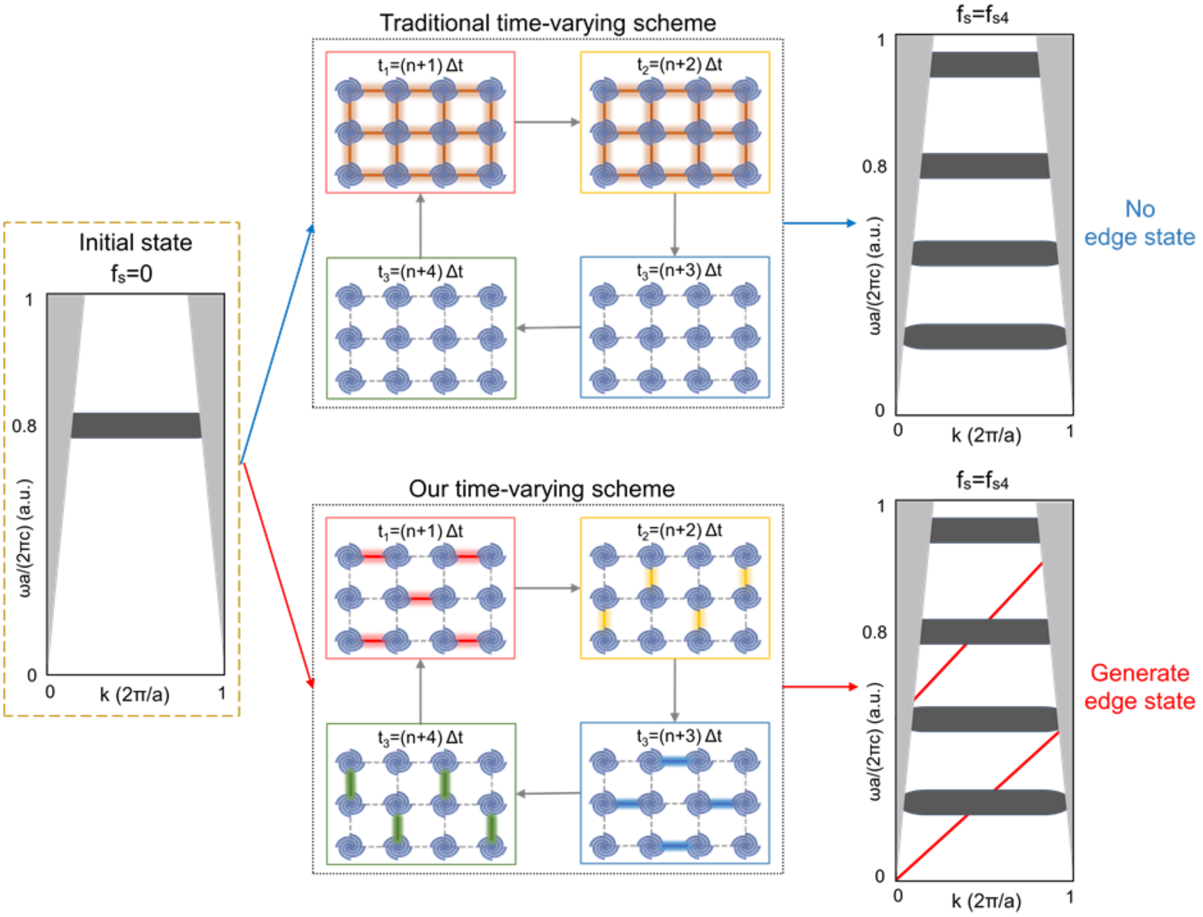

Fig. S13. The principle of edge state generation of the proposed topological metasurface.

In contrast, within our time-varying scheme, topological edge states (depicted by red line) can be observed between adjacent bulk-state regions at specific switching frequencies. This phenomenon can be explained through several aspects. Firstly, the time-driven Floquet protocol introduces periodic modulation, leading to the formation of new topological structures in the system's Floquet quasi- energy spectrum. Secondly, the specific switching frequency resonates with the system's eigen frequencies, thereby inducing localized edge state modes at the topological boundaries. Particularly, a distinct edge state emerges in each gap between adjacent bulk bands, a phenomenon fundamentally different from traditional modulation systems. A more comprehensive theoretical explanation of our time-varying scheme can be found in [Supplementary Materials Section 1]. These results indicate that harmonic bulk states can be generally generated in any time-varying wave system, whereas the Floquet topological states can only be generated in a specific time-varying scheme. Experimental results further validate our proposed method, which provides

new research avenues for exploring novel topological phase transitions and quantum transport phenomena, while also laying a theoretical foundation for the design of new quantum devices based on edge states.

Section 12: Robustness analysis based on synchronism and time jitter

In many systems such as wireless communications, digital signal processing, and timing controls, the synchronization effects between different units (or modules) during switching are crucial. Meanwhile, robustness is one of the most significant advantages of topological insulators. In this section, we primarily explore the robustness of our system by analyzing two key effects: time synchronization and time jitter.

Firstly, we define the time desynchronization parameter: within a cycle, when four different PIN diodes are turned on sequentially, the overall waveform of the square wave time-modulated signal between adjacent PIN diodes remains unchanged, with only a certain time error occurring at the preset rise time, as illustrated in Fig. S14a. In this example, we present the system analysis results at a switching frequency of 450 MHz. At this switching frequency, we gradually increase the time desynchronization and place a probe at a specific location along the metasurface propagation path to serve as the signal receiver. The output electric field spectrum is shown in Fig. S14b. Under 225 MHz single-tone signal excitation, we observe that even with varying degrees of time desynchronization, a strong signal response is achieved at both the fundamental and harmonic frequencies. To visually illustrate this robust transmission characteristic, we provide the near-field distribution results of the metasurface at the fundamental frequency, as well as the first and second harmonic frequencies in Fig. S14c, with the propagation path marked by a yellow dashed box. Notably, when the time desynchronization reaches 80 ps, which corresponds to more than 14.4% of the activation time of a single PIN diode within one switching cycle, the transmission performance is significantly reduced. However, below this value, the metasurface demonstrates a good robustness in propagation capability. In our experimental setup, all RF lines are of equal length, resulting in a time desynchronization ratio of less than 0.72%, which is far below the system's tolerance threshold; thus, our system maintains a high level of time synchronization.

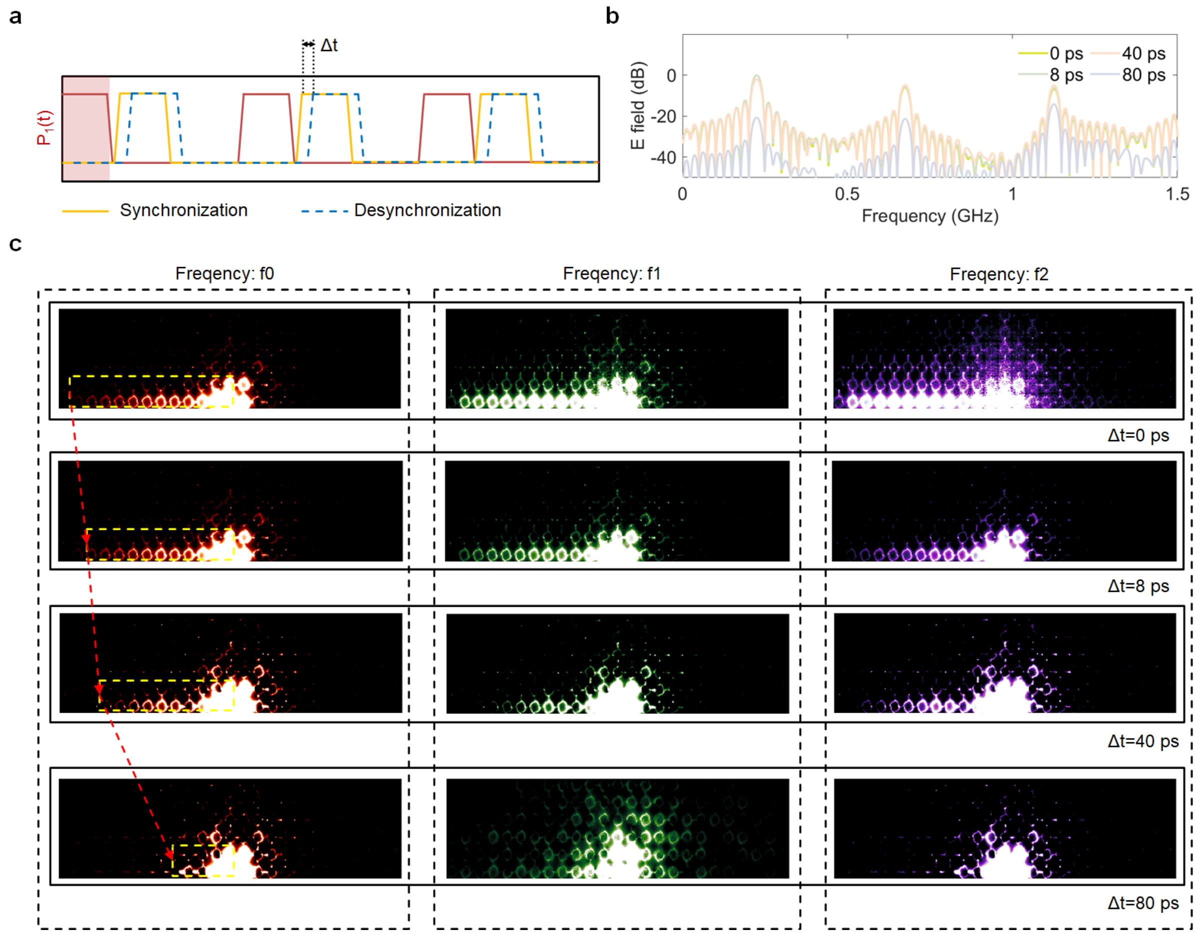

Fig. S14. Analysis of the propagation robustness of time-varying topological metasurfaces based on time synchronization discrepancies. a, Schematic illustration of time synchronization discrepancies, revealing the time delay in the activation of adjacent PIN diodes. **b,** Comparison of output electric fields under synchronized and time-discrepant conditions, specifically at time delays of 8 ps, 40 ps, and 80 ps. **c,** Results of near-field unidirectional propagation of the time-varying topological metasurface under both synchronized and time-discrepant conditions at fundamental, first-order, and second-order harmonic frequencies. It is observed that as the degree of time synchronization discrepancy increases, the propagation capability progressively diminishes.

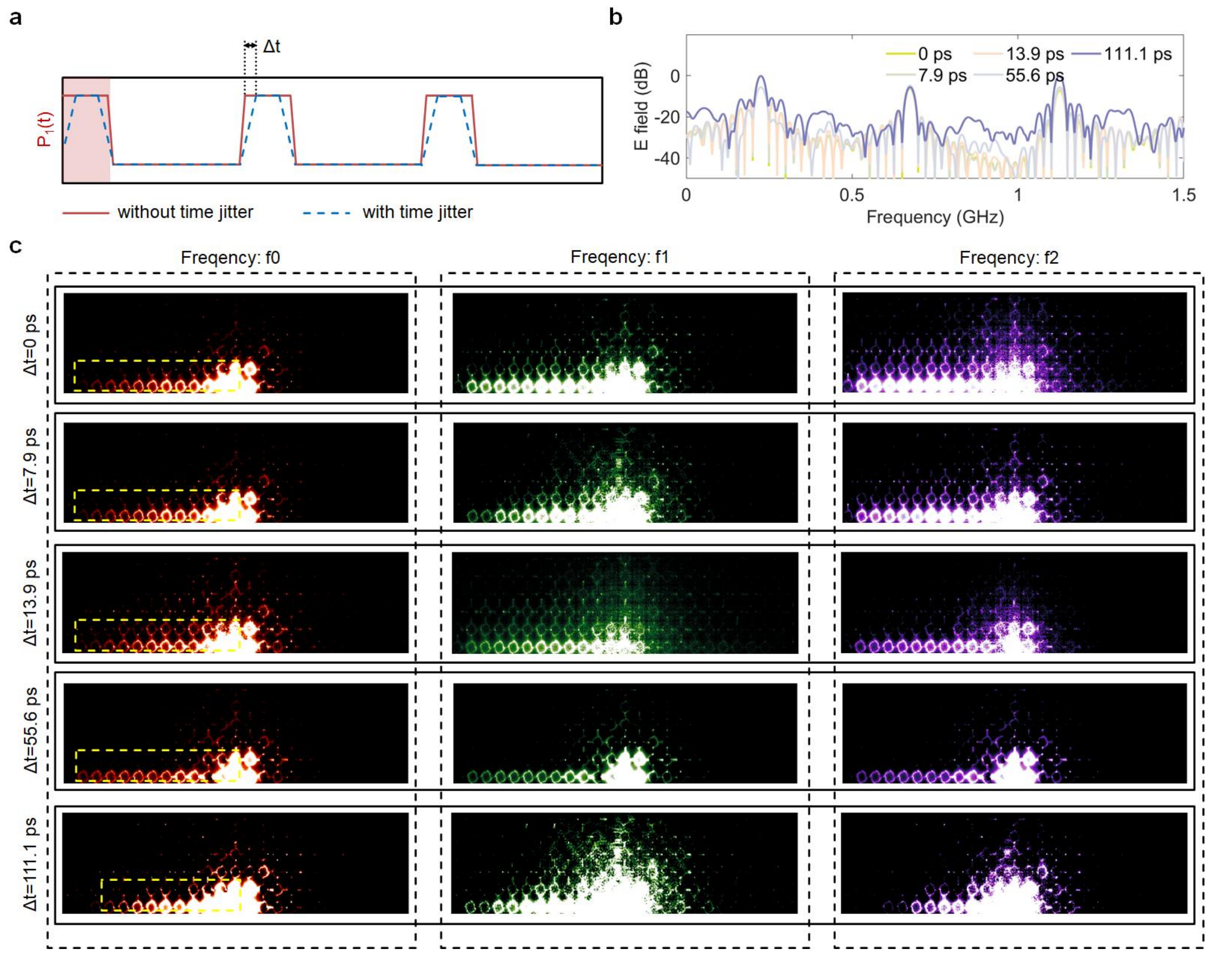

Fig. S15. Analysis of the propagation robustness of time-varying topological metasurfaces based on time jitter. **a,** Schematic illustration of time jitter, depicting the deviation of the rising and falling edges of a square wave signal along the time axis. **b,** Comparison of output electric fields under conditions of no time jitter and varying time jitter (specifically at 7.9 ps, 13.9 ps, 55.6 ps, and 111.1 ps). **c,** Results of near-field unidirectional propagation of the time-varying topological metasurface at fundamental, first-order, and second-order harmonic frequencies under both no time jitter and varying time jitter conditions (the same time delays as mentioned).

Secondly, regarding to the effect of time jitter, we define it as the deviation of the rising or falling edges of the square wave signal along the time axis. Ideally, the rising and falling edges of the square wave signal should occur at precise time points; however, in practical systems, various factors can induce slight time deviations in these edges. This time deviation is referred to as time jitter, as shown in Fig. S15a. Furthermore, we present both the output electric field spectrum distribution without time jitter and under various time jitter conditions (7.9 ps, 13.9 ps, 55.6 ps, and 111.1 ps) in Fig. S15b, along with the near-field distribution results at the fundamental frequency and the first and second harmonics in Fig. S15c. Even when time jitter is increased to a

moderately high value, the excellent robustness of the propagation characteristics remains intact. Overall, through the analysis of time desynchronization and time jitter effects, we further confirm the superior robustness of the programmable time-varying topological metasurface platform.

Section 13: Relationship between specific switching frequencies and SLSP resonator's resonance frequency

In this section, we explore the relationship between the system's intrinsic resonance frequency and the specific switching frequency required to achieve a topological phase transition. To facilitate a deeper analysis of this fundamental resonance phenomenon, we primarily focus on the prominent eigen frequency at 1.35 GHz of the SLSP resonator. As the switching frequency increases, the resonance frequency at f_r= 1.35 GHz of the SLSP resonator can induce chiral edge modes at specific switching frequencies, as illustrated in Fig. S16b. This phenomenon indicates a significant correlation between the specific switching frequencies and the resonance frequency of the SLSP resonator. We can express this relationship mathematically as follows (*53*):

$$f_{ss} = \frac{f_r}{k} \ (k = 1,2,3 \cdots) \tag{13.1}$$

where f_{ss} denotes the specific switching frequency and f_r signifies the resonance frequency of the SLSP resonator. However, it is important to emphasize that as the value of k increases, the intensity of the chiral edge modes excited at the corresponding frequencies gradually diminishes, as shown in Fig. S16a. Therefore, though theoretically there are infinitely many specific switching frequencies in the system, due to practical limitations, such as weak responses at $k > 7$ or unreachable high switching frequencies at $k = 1,2$ in our experiments, only specific switching frequencies at $k = 3,4,5,6,7$ are discussed in the main text to maintain consistency with the experiments.

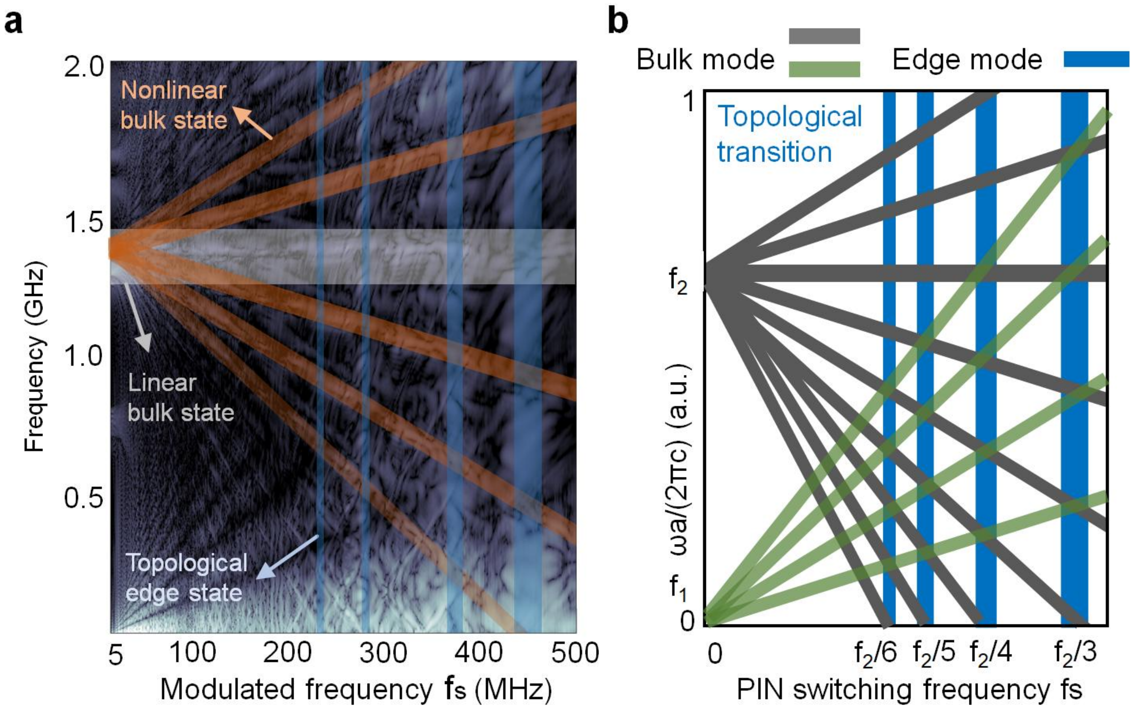

Fig. S16. Relationship between specific switching frequencies and the fundamental resonance of the SLSP resonator. a, As the switching frequency increases, topological transitions occur in the metasurface, where a series of harmonic bands (highlighted in brown) are observed experimentally at specific switching frequencies (i.e., transition frequencies) of 225 MHz, 275 MHz, 350 MHz, and 450 MHz. **b,** Topological phase transitions induced by the PIN switching frequency, where the Floquet harmonic bands originate from the resonance frequency of the SLSP resonator. With the increase of the switching frequency, at certain specific frequencies (namely one-sixth, one-fifth, one-fourth, and one-third of the resonance frequency), anomalous edge modes will emerge within the Floquet harmonic bandgap.

Moreover, as depicted in Fig. S5, our experimental approach also utilized 180 MHz as the switching frequency, successfully observing the related phenomenon. This experimental result further corroborates the theoretical relationship between the specific switching frequency and the resonance frequency of the system. The above comprehensive analysis of the relationship between the specific switching frequencies and the system's intrinsic resonance frequency provides a clearer theoretical foundation and experimental basis for understanding topological phase transitions in our programmable time-varying topological metasurface platform.

Section 14: Stability analysis based on phase matching

A well-known prerequisite for achieving an effective frequency conversion process is the phase matching of the interacting waves. Therefore, in this section, we will explore the phase matching conditions necessary for efficient Floquet harmonic generation. A special feature of time-varying Floquet systems is that the spectrum along the frequency axis has periodicity, i.e., the edge state modes within different band gaps are Floquet replicas of the same edge state dispersion curve. As a result, the edge state modes across different band gaps have a high degree of overlap, thus ensuring a high level of phase matching. To show this, we illustrate in Fig. S17a the band structure of the metasurface at a switching frequency of 450 MHz, focusing on the first two band gaps as an example, specifically in the ranges of 0-450 MHz and 450-900 MHz. After shifting and comparing the edge state curves (considering the significant differences in mode intensity between the first and second band gaps) in the two band gaps, we can see that the edge state curves within the two gaps have a high level overlap, indicating that our system possesses a high degree of phase matching characteristics.

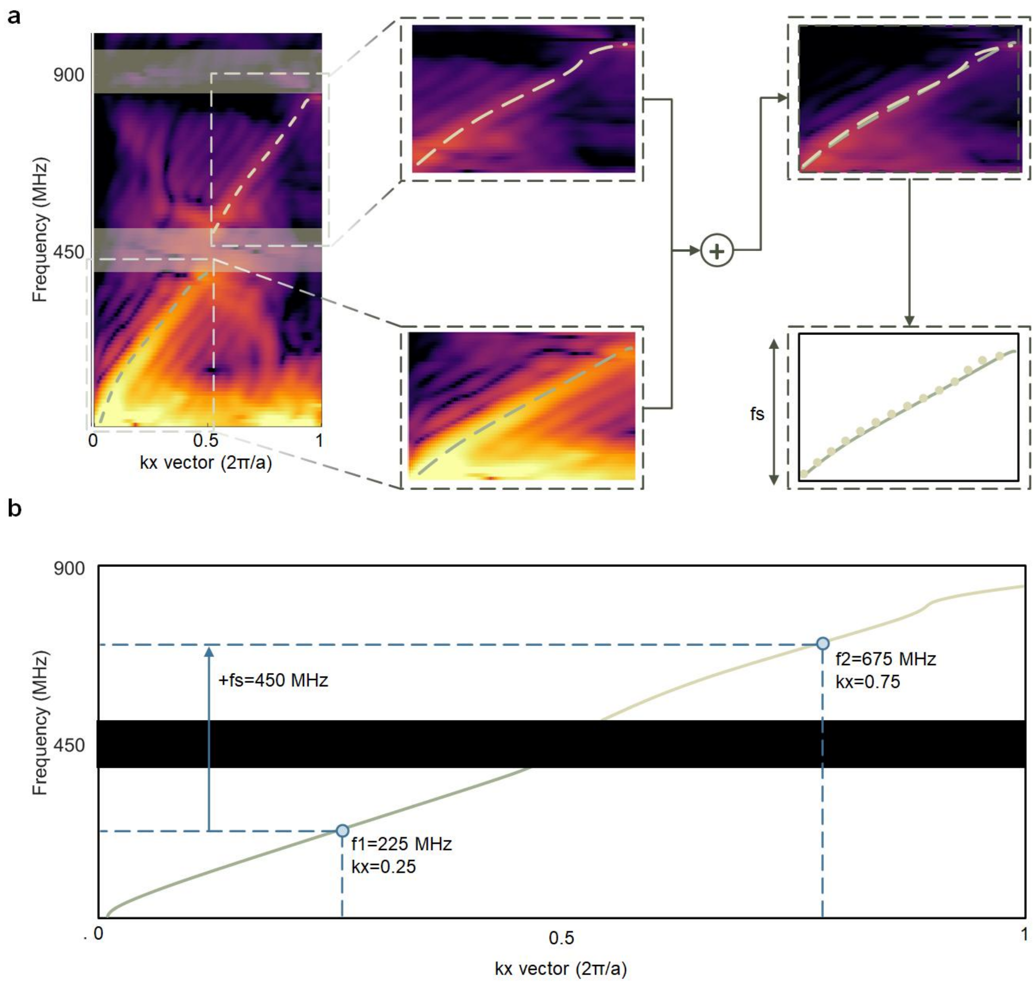

Fig. S17. Analysis of Phase Matching Conditions. a, Comparison of the edge state modes in the first and second band gaps. **b,** Phase matching of the Floquet harmonic generation in a time-varying topological metasurface platform at a switching frequency of 450 MHz.

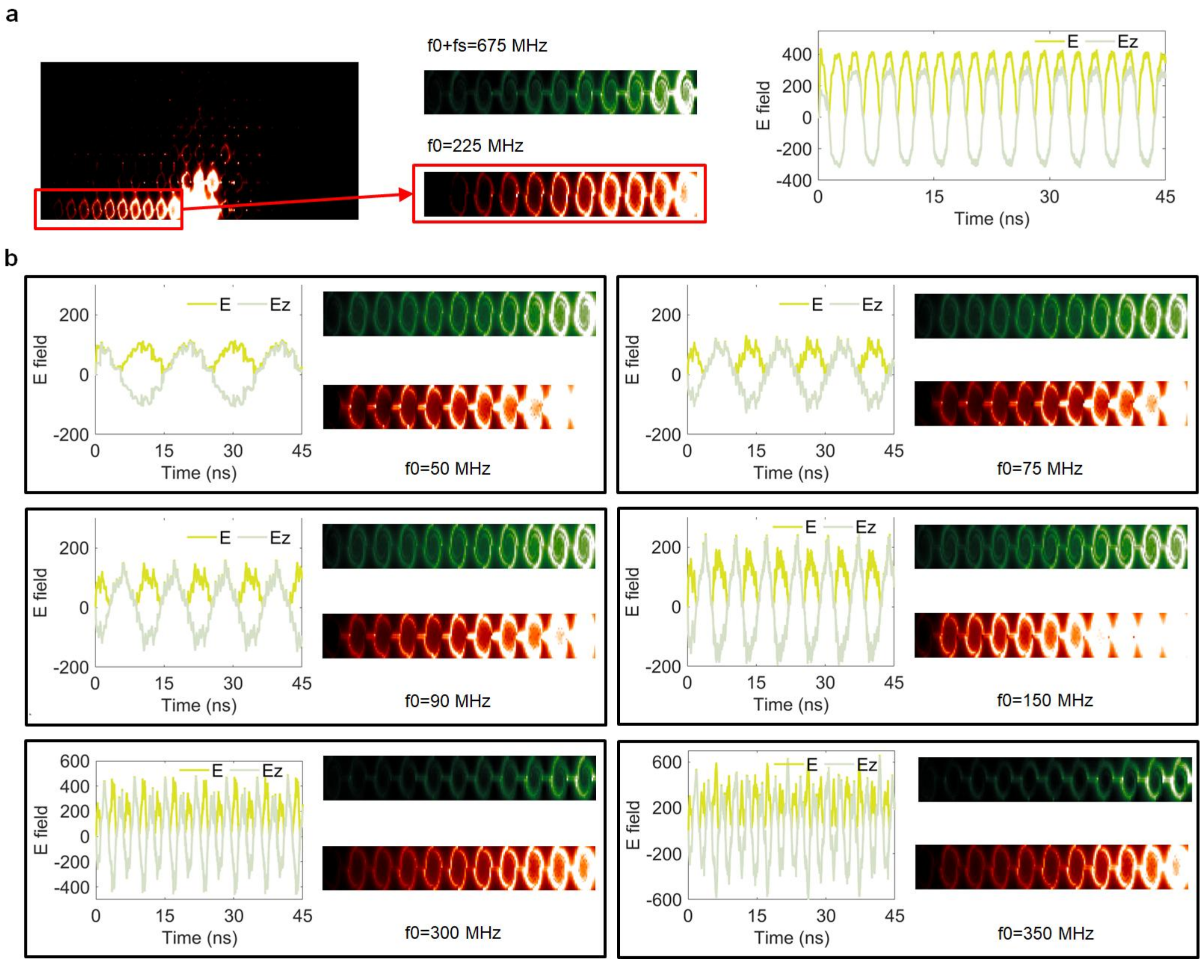

Fig. S18. Analysis of the propagation stability of Floquet harmonic waves under different excitation frequency $\boldsymbol{f_0}$**. a,** Near-field distribution of the fundamental frequency and the first harmonic, along with the corresponding time-dependent trend of the electric field, under the excitation of a 225 MHz monochromatic signal. **b,** Similar as in **a** but under the excitation of monochromatic signals at various frequencies (including 50 MHz, 75 MHz, 90 MHz, 150 MHz, 300 MHz, and 350 MHz).

To further validate this, we present the near-field distributions of the fundamental frequency and the first harmonic frequency under various excitation frequency of f_0 (including 50 MHz, 75 MHz, 90 MHz, 150 MHz, 225 MHz, 300 MHz, and 350 MHz, i.e., spanning roughly the whole band gap), along with the corresponding time-varying electric field curves, as shown in Fig. S18.

As illustrated in Fig. S18b, regardless of how the excitation frequency of the single-tone signal in the first band gap varies, sizeable first harmonic could all be generated with coherent stable periodic oscillations over time. The above results illustrate that the mechanism of Floquet replicas of the edge state modes in different band gaps ensures a high level of phase matching for the Floquet harmonic generation in our time-varying topological metasurface platform.

REFERENCES AND NOTES

1. S. Yin, E. Galiffi, A. Alù, Floquet metamaterials. *eLight* **2**, 8 (2022).

2. J. E. Moore, The birth of topological insulators. *Nature* **464**, 194–198 (2010).

3. T. Kitagawa, E. Berg, M. Rudner, E. Demler, Topological characterization of periodically driven quantum systems. *Phys. Rev. B* **82**, 235114 (2010).

4. M. Z. Hasan, C. L. Kane, Colloquium: Topological insulators. *Rev. Mod. Phys.* **82**, 3045–3067 (2010).

5. Y. Yang, Z. Gao, H. Xue, L. Zhang, M. He, Z. Yang, R. Singh, Y. Chong, B. Zhang, H. Chen, Realization of a three-dimensional photonic topological insulator. *Nature* **565**, 622–626 (2019).

6. H. Jia, R. Zhang, W. Gao, Q. Guo, B. Yang, J. Hu, Y. Bi, Y. Xiang, C. Liu, S. Zhang, Observation of chiral zero mode in inhomogeneous three-dimensional Weyl metamaterials. *Science* **363**, 148–151 (2019).

7. S. Kruk, A. Poddubny, D. Smirnova, L. Wang, A. Slobozhanyuk, A. Shorokhov, I. Kravchenko, B. Luther-Davies, Y. Kivshar, Nonlinear light generation in topological nanostructures. *Nat. Nanotechnol.* **14**, 126–130 (2019).

8. D. J. Thouless, M. Kohmoto, M. P. Nightingale, M. den Nijs, Quantized Hall conductance in a two-dimensional periodic potential. *Phys. Rev. Lett.* **49**, 405–408 (1982).

9. K. v. Klitzing, G. Dorda, M. Pepper, New method for high-accuracy determination of the fine-structure constant based on quantized Hall resistance. *Phys. Rev. Lett.* **45**, 494–497 (1980).

10. K. Fang, Z. Yu, S. Fan, Realizing effective magnetic field for photons by controlling the phase of dynamic modulation. *Nat. Photonics* **6**, 782–787 (2012).

11. M. S. Rudner, N. H. Lindner, E. Berg, M. Levin, Anomalous edge states and the bulk-edge correspondence for periodically driven two-dimensional systems. *Phys. Rev. X* **3**, 031005 (2013).

12. M. C. Rechtsman, J. M. Zeuner, Y. Plotnik, Y. Lumer, D. Podolsky, F. Dreisow, S. Nolte, M. Segev, A. Szameit, Photonic Floquet topological insulators. *Nature* **496**, 196–200 (2013).

13. G. G. Pyrialakos, J. Beck, M. Heinrich, L. J. Maczewsky, N. V. Kantartzis, M. Khajavikhan, A. Szameit, D. N. Christodoulides, Bimorphic Floquet topological insulators. *Nat. Mater.* **21**, 634–639 (2022).

14. S. Stützer, Y. Plotnik, Y. Lumer, P. Titum, N. H. Lindner, M. Segev, M. C. Rechtsman, A. Szameit, Photonic topological Anderson insulators. *Nature* **560**, 461–465 (2018).

15. D. Leykam, Y. D. Chong, Edge solitons in nonlinear-photonic topological insulators. *Phys. Rev. Lett.* **117**, 143901 (2016).

16. D. Leykam, M. C. Rechtsman, Y. D. Chong, Anomalous topological phases and unpaired dirac cones in photonic floquet topological insulators. *Phys. Rev. Lett.* **117**, 013902 (2016).

17. T. Biesenthal, L. J. Maczewsky, Z. Yang, M. Kremer, M. Segev, A. Szameit, M. Heinrich, Fractal photonic topological insulators. *Science* **376**, 1114–1119 (2022).

18. M. Hafezi, S. Mittal, J. Fan, A. Migdall, J. M. Taylor, Imaging topological edge states in silicon photonics. *Nat. Photonics* **7**, 1001–1005 (2013).

19. S. Mittal, V. V. Orre, G. Zhu, M. A. Gorlach, A. Poddubny, M. Hafezi, Photonic quadrupole topological phases. *Nat. Photonics* **13**, 692–696 (2019).

20. M. Hafezi, E. A. Demler, M. D. Lukin, J. M. Taylor, Robust optical delay lines with topological protection. *Nat. Phys.* **7**, 907–912 (2011).

21. Y. Ao, X. Hu, Y. You, C. Lu, Y. Fu, X. Wang, Q. Gong, Topological phase transition in the non-Hermitian coupled resonator array. *Phys. Rev. Lett.* **125**, 013902 (2020).

22. G. Q. Liang, Y. D. Chong, Optical resonator analog of a two-dimensional topological insulator. *Phys. Rev. Lett.* **110**, 203904 (2013).

23. S. Mittal, J. Fan, S. Faez, A. Migdall, J. M. Taylor, M. Hafezi, Topologically robust transport of photons in a synthetic gauge field. *Phys. Rev. Lett.* **113**, 087403 (2014).

24. S. Mittal, V. V. Orre, D. Leykam, Y. D. Chong, M. Hafezi, Photonic anomalous quantum Hall effect. *Phys. Rev. Lett.* **123**, 043201 (2019).

25. M. A. Bandres, S. Wittek, G. Harari, M. Parto, J. Ren, M. Segev, D. N. Christodoulides, M. Khajavikhan, Topological insulator laser: Experiments. *Science* **359**, eaar4005 (2018).

26. G. Harari, M. A. Bandres, Y. Lumer, M. C. Rechtsman, Y. D. Chong, M. Khajavikhan, D. N. Christodoulides, M. Segev, Topological insulator laser: Theory. *Science* **359**, eaar4003 (2018).

27. R. Fleury, A. B. Khanikaev, A. Alù, Floquet topological insulators for sound. *Nat. Commun.* **7**, 11744 (2016).

28. A. Darabi, X. Ni, M. Leamy, A. Alù, Reconfigurable Floquet elastodynamic topological insulator based on synthetic angular momentum bias. *Sci. Adv.* **6**, eaba8656 (2020).

29. K. Fang, S. Fan, Controlling the flow of light using the inhomogeneous effective gauge field that emerges from dynamic modulation. *Phys. Rev. Lett.* **111**, 203901 (2013).

30. M. Li, X. Ni, M. Weiner, A. Alù, A. B. Khanikaev, Topological phases and nonreciprocal edge states in non-Hermitian Floquet insulators. *Phys. Rev. B* **100**, 045423 (2019).

31. A. Nagulu, X. Ni, A. Kord, M. Tymchenko, S. Garikapati, A. Alù, H. Krishnaswamy, Chip-scale Floquet topological insulators for 5G wireless systems. *Nat. Electron.* **5**, 300–309 (2022).

32. J. You, Q. Ma, L. Zhang, C. Liu, J. Zhang, S. Liu, T. Cui, Electromagnetic metamaterials: From classical to quantum. *Electromagn. Sci.* **1**, 0010051 (2023).

33. T. J. Cui, M. Q. Qi, X. Wan, J. Zhao, Q. Cheng, Coding metamaterials, digital metamaterials and programmable metamaterials. *Light Sci. Appl.* **3**, e218 (2014).

34. J. W. You, Q. Ma, Z. Lan, Q. Xiao, N. C. Panoiu, T. J. Cui, Reprogrammable plasmonic topological insulators with ultrafast control. *Nat. Commun.* **12**, 5468 (2021).

35. C. Liu, Q. Ma, Z. J. Luo, Q. R. Hong, Q. Xiao, H. C. Zhang, L. Miao, W. M. Yu, Q. Cheng, L. Li, T. J. Cui, A programmable diffractive deep neural network based on a digital-coding metasurface array. *Nat. Electron.* **5**, 113–122 (2022).

36. L. Zhang, M. Z. Chen, W. Tang, J. Y. Dai, L. Miao, X. Y. Zhou, S. Jin, Q. Cheng, T. J. Cui, A wireless communication scheme based on space- and frequency-division multiplexing using digital metasurfaces. *Nat. Electron.* **4**, 218–227 (2021).

37. J. W. You, Z. Lan, Q. Ma, Z. Gao, Y. Yang, F. Gao, M. Xiao, T. J. Cui, Topological metasurface: From passive toward active and beyond. *Photonics Res.* **11**, B65–B102 (2023).

38. Q. Ma, W. Gao, Q. Xiao, L. Ding, T. Gao, Y. Zhou, X. Gao, T. Yan, C. Liu, Z. Gu, X. Kong, Q. H. Abbasi, L. Li, C.-W. Qiu, Y. Li, T. J. Cui, Directly wireless communication of human minds via non-invasive brain-computer-metasurface platform. *eLight* **2**, 11 (2022).

39. X. Gao, Q. Ma, Z. Gu, W. Y. Cui, C. Liu, J. Zhang, T. J. Cui, Programmable surface plasmonic neural networks for microwave detection and processing. *Nat. Electron.* **6**, 319–328 (2023).

40. A. Pors, E. Moreno, L. Martin-Moreno, J. B. Pendry, F. J. Garcia-Vidal, Localized spoof plasmons arise while texturing closed surfaces. *Phys. Rev. Lett.* **108**, 223905 (2012).

41. P. A. Huidobro, X. Shen, J. Cuerda, E. Moreno, L. Martin-Moreno, F. J. Garcia-Vidal, T. J. Cui, J. B. Pendry, Magnetic localized surface plasmons. *Phys. Rev. X* **4**, 021003 (2014).

42. M. S. Rudner, N. H. Lindner, Band structure engineering and non-equilibrium dynamics in Floquet topological insulators. *Nat. Rev. Phys.* **2**, 229–244 (2020).

43. S. Yao, Z. Yan, Z. Wang, Topological invariants of Floquet systems: General formulation, special properties, and Floquet topological defects. *Phys. Rev. B* **96**, 195303 (2017).

44. W. Y. Cui, J. Zhang, Y. Luo, X. Gao, T. J. Cui, Dynamic switching from coherent perfect absorption to parametric amplification in a nonlinear spoof plasmonic waveguide. *Nat. Commun.* **15**, 2824 (2024).

45. J. W. You, H. G. Wang, J. F. Zhang, S. R. Tan, T. J. Cui, Accurate numerical method for multipactor analysis in microwave devices. *IEEE Trans. Electron Devices* **61**, 1546–1552 (2014).

46. J. W. You, J. F. Zhang, W. X. Jiang, H. F. Ma, W. Z. Cui, T. J. Cui, Accurate analysis of finite-volume lumped elements in metamaterial absorber design. *IEEE Trans. Microw. Theory Tech.* **64**, 1966–1975 (2016).

47. J. W. You, H. G. Wang, J. F. Zhang, S. R. Tan, T. J. Cui, Accurate numerical analysis of nonlinearities caused by multipactor in microwave devices. *IEEE Microw. Wirel. Compon. Lett.* **24**, 730–732 (2014).

48. J. W. You, H. G. Wang, J. F. Zhang, W. Z. Cui, T. J. Cui, The conformal TDFIT-PIC method using a new extraction of conformal information (ECI) technique. *IEEE Trans. Plasma Sci.* **41**, 3099–3108 (2013).

49. J. W. You, S. R. Tan, T. J. Cui, Novel adaptive steady-state criteria for finite-difference time-domain method. *IEEE Trans. Microw. Theory Tech.* **62**, 2849–2858 (2014).

50. J. W. You, J. F. Zhang, W. H. Gu, W. Z. Cui, T. J. Cui, Numerical analysis of passive intermodulation arisen from nonlinear contacts in HPMW devices. *IEEE Trans. Electromagn. Compat.* **60**, 1470–1480 (2018).

51. Z. Lou, X. Wu, J. Hou, J. Zhang, J. You, T. Cui, Numerical modelling of dynamic electromagnetic problems based on the time-domain finite integration technique. *Electronics* **11**, 3912 (2022).

52. L. Chen, J. W. You, X. W. Wu, Q. Xiao, Z. Gu, S. L. Qin, T. J. Peng, Q. Ma, Y. Q. Mao, J. N. Zhang, T. J. Cui, Accurate modeling of time-varying lumped elements in space-time metasurfaces. *IEEE Trans. Antennas Propag.* **73**, 3849–3862 (2025).

53. J. D. Jackson, R. F. Fox, Classical Electrodynamics, 3rd ed. *Am. J. Phys.* **67**, 841–842 (1999).
